# Direct Preparation of *N*-Substituted Pyrazoles from Primary Aliphatic or Aromatic Amines

**DOI:** 10.1021/acs.joc.1c00606

**Published:** 2021-07-01

**Authors:** Nurbey Gulia, Marcin Małecki, Sławomir Szafert

**Affiliations:** Faculty of Chemistry, University of Wrocław, 14 F. Joliot-Curie, 50-383 Wrocław, Poland

## Abstract

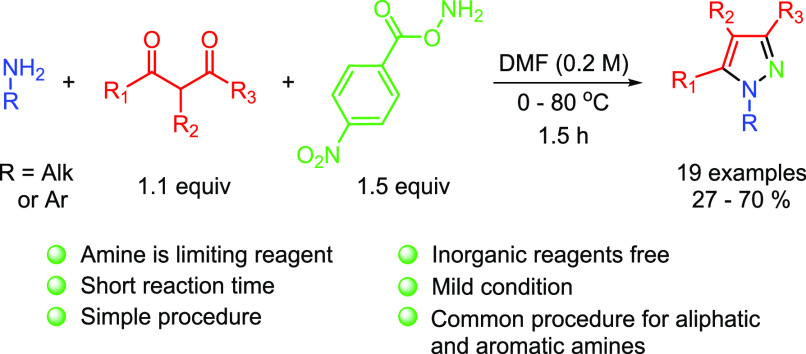

Despite a large number of synthesis procedures for pyrazoles known today, those directly employing primary amines as substrates are rare. Herein, we report an original method for the preparation of *N*-alkyl and *N*-aryl pyrazoles from primary aliphatic or aromatic amines as a limiting reagent of the reaction. The protocol utilizes no inorganic reagents and requires a short reaction time, mild conditions, and the use of structurally simple and commercially available starting reagents. During this study, pyrazoles containing a wide variety of *N*-substituents were obtained using the same procedure for both aliphatic and aromatic amines.

## Introduction

Pyrazoles can be obtained by numerous synthetic methods,^1−5^ but their versatile applicability as pharmaceuticals,^6,7^ crop protection chemicals,^8,9^ building blocks for organic and inorganic chemistry^10,11^ still propels research to develop new synthetic methodologies. Over the past decade, a new specific application has appeared for *N*-substituted pyrazoles, i.e., as a metal-coordinating directing group for transition-metal-catalyzed reactions.^12−18^ A large number of such chemical transformations significantly increase interest in pyrazole-functionalized molecules. Preparation of *N*-substituted pyrazoles from primary amines as a limiting reagent and source of *N*-substituent gives a wide variety of potential products that might be further functionalized.^15^

**Scheme 1:**
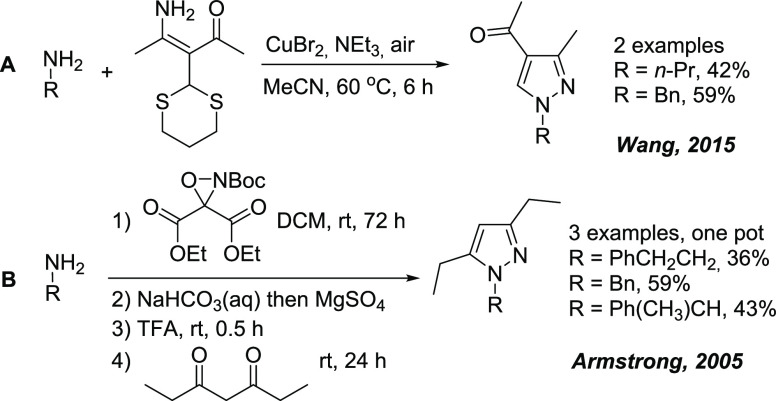
Pyrazoles from Aliphatic Amines as a Limiting Reagent

In contrast to *N*-alkyl indazoles for which synthetic methodology starting from primary aliphatic amines is well-known and broadly used,^19−21^*N*-alkyl pyrazoles are usually synthesized from difficult to handle hydrazines or hydrazine derivatives.^2−4,22−24^ With a great variety of synthetic methods for *N*-alkyl pyrazoles, there are just two reports describing primary aliphatic amine as a limiting reagent that introduces an *N*-linked substituent into a product’s structure (Scheme 1).^25,26^ The known methods are limited because of multistep functionalizations of diketone (**A**) or amination reagent preparation (**B**). The drawback of the methodologically elegant reaction **B** is, moreover, a complicated and time-consuming procedure (Scheme 1).

Electrophilic amination of primary aliphatic amines is a well-known strategy for the preparation of hydrazines.^27−29^ However, in all cases, a large excess of the amine is required due to hydrazine’s enhanced nucleophilicity relative to the corresponding origin amine.^30^ This problem might be solved by utilizing *N*-protected electrophilic amination reagents that form hydrazines that are stable under reaction conditions, but this requires a further deprotection step.^25,31,32^ Transformation with commercially available amination reagents that form unprotected hydrazine has practical advantages. Here we report a fast and straightforward method for the preparation of pyrazoles from primary aliphatic and aromatic amines as limiting reagents using bench-stable, commercially available amination reagent.

## Results and Discussion

Initially, we tested reagents **R1**, **R2**, and **R4**, and we found that only **R1** gives the desired product under initial conditions. Solvent, temperature, time, and proportion of reagents optimization with **R1** allowed obtaining the desired *N*-alkyl pyrazole **1a** with 44% isolated yield (Table 1, for the full table, see Table S1). Adding all three reagents simultaneously at 0 °C followed by their heating is critical to obtain the desired product with a reproducible yield. Minor changes in the reaction temperature or equivalents of reagents **R1** and **1** slightly reduce the yield (Table 1, entries 3–7). The addition of a stoichiometric amount of water, acetic acid, or weak basic salts has a minor effect on yield, showing that the reaction is not promoted by this species, and it is not sensitive to them (Table 1, entries 8–12). The presence of diisopropylethylamine (DIPEA) decreases the yield of **1a**, which is probably an effect of an **R1** consumption for a side formation of 1,1-diisopropyl-1-ethylhydrazonium salt (entry 12). Alternative amination reagents **R2**–**R6** were tried under optimized conditions (Table 1, entries 13–17). The product was obtained in 23%, 41%, and 53% yields for **R2**, **R3**, and **R6**, respectively, and was not observed (^1^H NMR and GC–MS) for reactions conducted with **R4** and **R5**.

**Table 1:**
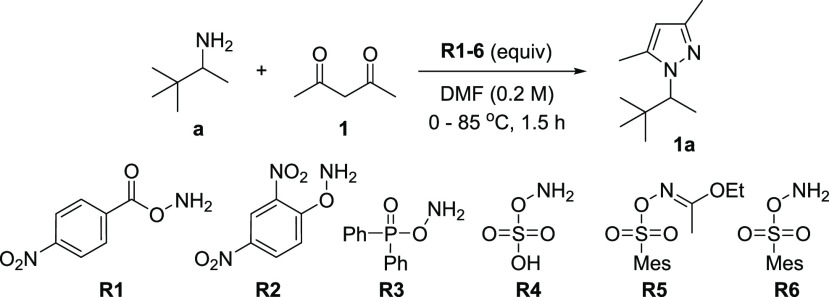
Reaction Conditions and Amination Reagent Optimization^a^

entry	**R1**–**6** [equiv]	additive [1.0 equiv]	temp [°C]	yield^b^ [%]
1	**R1** [1.5]		0–85	54, 53,^e^ 44^f^
2^c^	**R1** [1.5]		0–85	17
3	**R1** [1.5]		0–110	49
4	**R1** [1.5]		0–50	41
5	**R1** [1.1]		0–85	47
6	**R1** [1.7]		0–85	48
7^d^	**R1** [1.5]		0–85	48
8	**R1** [1.5]	H_2_O	0–85	51
9	**R1** [1.5]	AcOH	0–85	51
10	**R1** [1.5]	AcONa	0–85	53
11	**R1** [1.5]	K_2_CO_3_	0–85	56
12	**R1** [1.5]	DIPEA	0–85	46
13	**R2** [1.5]		0–85	23^e^
14	**R3** [1.5]		0–85	41^e^
15	**R4** [1.5]		0–85	0^e^
16	**R5** [1.5]		0–85	0^e^
17	**R6**^g^ [1.5]		0–85	53^e^

a Reactions were carried out using **a** (0.2 mmol, 1.0 equiv), **1** (0.22 mmol, 1.1 equiv), **R1**–**6** (1.1–1.7 equiv), and DMF (1.0 mL, 0.2 M) at a given temperature under air.

b GC yield.

c **1** was added after the mixture was heated to 80 °C.

d 0.3 mmol (1.5 equiv) of **1** was added.

e NMR yield.

f Isolated yield.

g Wet **R6** was used.

**Scheme 2:**
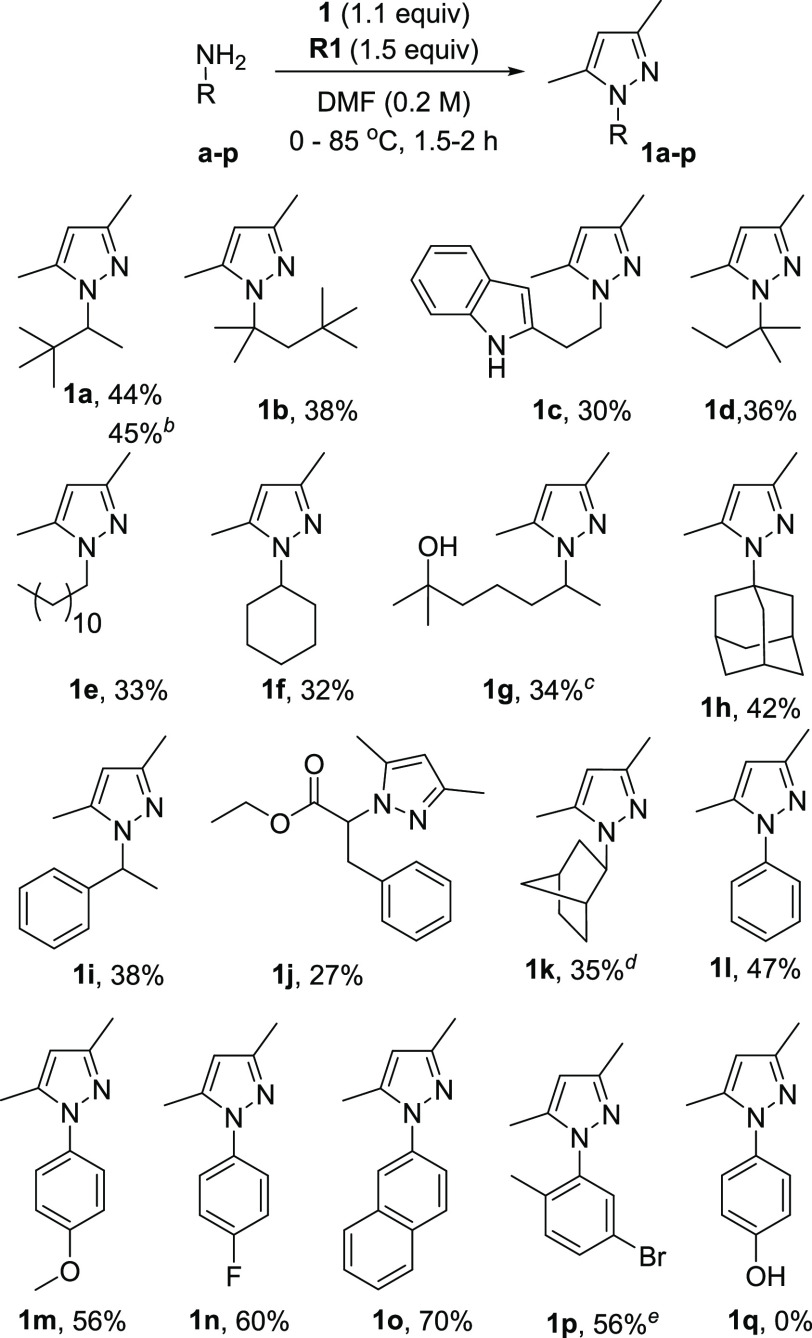
Scope of Amines The yields refer to the isolated products. Reactions were carried out using amines **a–q** (1.0 mmol), **1** (1.1 mmol), **R1** (1.5 mmol), and DMF (5.0 mL) at 0–85 °C in 1.5–2.0 h under air unless otherwise given. The experiment was conducted on a 3.0 mmol scale to give 0.243 g (1.35 mmol) of the **1a**. The experiment was conducted on a 5.0 mmol scale to give 383 mg (1.71 mmol) of the **1g**. The experiment was conducted on a 0.5 mmol scale. The reaction was carried out for 16 h at 80 °C.

A series of primary aliphatic and aromatic amines were reacted under optimal conditions yielding corresponding pyrazoles (Scheme 2). Reactions were typically carried out on a 1 mmol scale. It was found that aliphatic amines with quaternary carbon or sterically hindered tertiary carbon adjacent to the amine group gave around 40% yields (**1a**, **1b**, **1d**, **1h**, and **1i**), whereas amines with neighboring secondary or less hindered tertiary carbon gave yields close to 30% (**1c**, **1e**, **1g**, **1j**, and **1k**). Aromatic amines reacted under the same condition and gave pyrazoles **1l**–**p** generally with higher yields (47–70%) than aliphatic amines. Compounds with electron-donating and withdrawing substituent at phenyl ring (**1m** and **1n**) and substituent in *ortho* position to an amine group (**1p**) were successfully obtained. Besides ester, methoxy, and haloarene functionalities, the reaction tolerates unprotected N–H of indole and aliphatic O–H group. However, the target product was not observed in the presence of unprotected phenol (**1q**), probably due to competitive *O*-amination. Syntheses of **1a** and **1g** were conducted on a 3.0 and 5.0 mmol scale, respectively, to underline the method’s synthetic applicability. The structure of **1o** was confirmed by X-ray single-crystal analysis (Scheme 4).

**Scheme 3:**
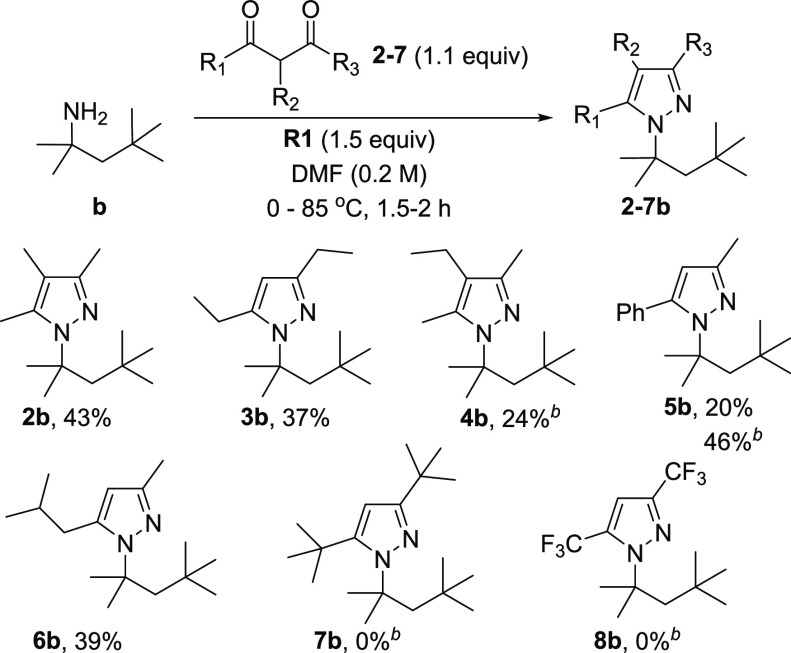
Scope of Diketones The yields refer to the isolated products. Reactions were carried out using amine **b** (1.0 mmol), diketones **2**–**8** (1.1 mmol), **R1** (1.5 mmol), and DMF (5.0 mL) at 0–85 °C for 1.5 h under air unless otherwise given. The experiment was conducted on a 0.5 mmol scale for **4b** and a 0.2 mmol for **5b** and **7**–**8b** with 5.0 equiv of diketones.

**Scheme 4:**
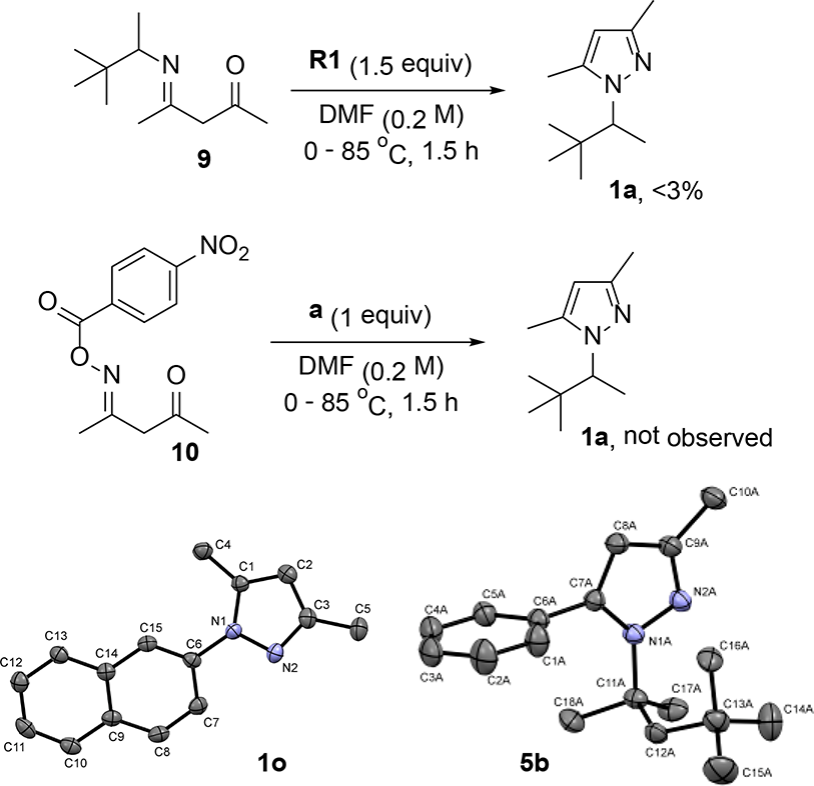
Additional Experiments and X-ray Structures The reactions were carried out using **9** (0.1 mmol, 1.0 equiv), **R1** (1.1 equiv), and DMF (0.5 mL); **10** (0.12 mmol, 1.2 equiv), **a** (1.0 equiv), and DMF (0.5 mL), at a given temperature under air. ORTEP view of crystal structures of **1o** (CCDC 2019272) and **5b** (CCDC 2019718). Thermal ellipsoids are drawn to encompass 50% probability level.

Reactivity of a series of diketones was tested in reaction with *tert*-octylamine, and it was found that it strongly depends on the electronic and steric properties of diketone’s substituents (Scheme 3). 3,5-Dialkyl and 3,4,5-trialkyl pyrazoles (**2b**, **3b**, and **6b**) were obtained in 37–43% yields, whereas **4b** in only 24% even with 5.0 equiv of **b**. Reaction conducted with diketone containing aryl substituent **5** under standard conditions gave a 20% yield. However, the use of 5.0 equiv of **5** instead of standard 1.1 allowed for **5b** with a 46% yield. Sterically hindered **7** and electron-deficient **8** did not react under the standard conditions and when they were used in 5.0 equiv excess (**7b** and **8b**). Interestingly, for reactions with diketones **5** and **6**, only one isomer of pyrazoles **5b** or **6b** was obtained. The other was observed in the crude reaction mixture by GC–MS in a very small amount, but they were not isolated in both cases. The structure of the obtained isomer **5b** was confirmed by X-ray single-crystal analysis (Scheme 4).

**Scheme 5:**
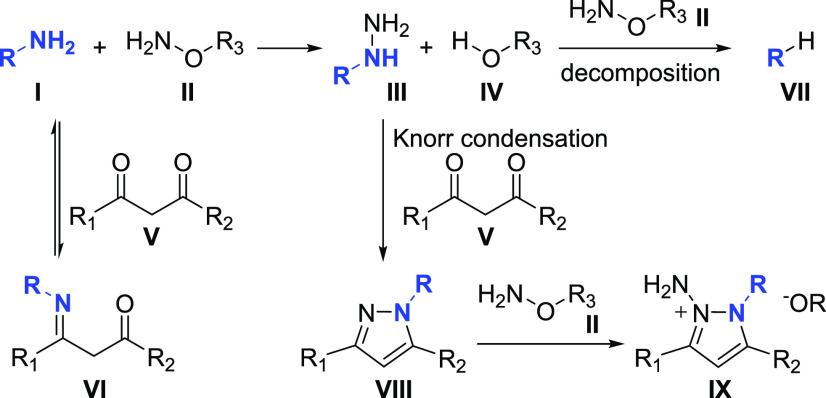
Proposed Reaction Pathway and Side Product Formation

Additional experiments conducted for **9** and **10** showed that both compounds are not intermediates in the reaction (Scheme 4). This and selectivity of formed products from unsymmetrical diketones (Scheme 3, **5b** and **6b**) that indicate a nucleophilic attack of prior formed hydrazine on less hindered carbonyl gave reason to believe that the reaction starts from a nucleophilic attack of amine on **II** (Scheme 5). Delivered during this step, hydrazine **III** is trapped by diketone **V** before the next competitive attack on **II**. It is also consistent with the fact that the addition of diketone **1** with a few minutes delay caused a significantly lower yield (17%, Table 1, entry 2). Relatively strong *p*-nitrobenzoic acid that forms in the first step from **R1** catalyzes the formation of hydrazone followed by heterocyclization to give pyrazole in Knorr condensation reaction. The observed moderate yield of the reaction explains the formation of side products **VI**, **VII**, and **IX** (Scheme 5). GC–MS of a crude mixture of **1a** indicated corresponding imine **VI**, and the product of hydrazine decomposition **VII** was observed by the same method running reaction without diketone (see Supporting Information). The product of amination of pyrazole **IX** was not detected in reaction mixtures. However, deprotonated pyrazole efficiently reacts with **R1**,^33^ and the addition of the next portion of amination reagent **II** decreases the yield of **VIII**, pointing to the formation of **IX** (see Table S1, entries 5 and 9).

In contrast to the multistep method of pyrazole synthesis from amines, which in showed examples gave comparable overall yields (Scheme 1, B),^25^ the presented method is characterized by a short time and a simple procedure that delivers products with less effort. In this context, the availability of **R1** amination reagent from commercial sources offers an extra advantage over the requiring five-step synthesis of oxaziridine (15% overall yield). Alternatively, a known two-step large-scale synthesis allows to obtain **R1** from basic reagents (81% overall yield).^33^

## Conclusions

In conclusion, we have developed a new method for the preparation of *N*-alkyl and *N*-aryl substituted pyrazoles directly from primary aliphatic or aromatic amines and diketones, applying readily accessible from commercial sources electrophilic amination reagent **R1**. Despite the modest yields in some cases, the use of an amine as the limiting reagent, the absence of metals, short reaction times, and a simple procedure makes this method practical for functionalizing amines, giving pyrazoles containing a wide variety of *N*-substituents.

## Experimental Section

### General Information

All reactions were carried out and purified using standard, commercially available glassware. Chemicals for reactions, workup, and chromatography were reagent grade or ACS grade and were used as received. Silica gel 60 Å 0.04–0.06 mm (Macherey Nagel), Al_2_O_3_, basic/neutral alumina, Brockmann grade I, 60 mesh (Alfa Aesar), was used for product purifications. ^1^H NMR and ^13^C NMR spectra were recorded using a 500 MHz Bruker Avance spectrometer with an inverse broad-band probe. For all ^1^H NMR spectra, the chemical shifts are given in ppm relative to the solvent residual peaks (CDCl_3_, ^1^H = 7.26 ppm, ^13^C = 77.16 ppm). Coupling constants are given in hertz (Hz). ^13^C NMR spectra were measured with proton decoupling. HRMS spectra were recorded using Bruker Apex ultra FT-ICR (ESI) or Shimadzu q-TOF LCMS 9030 with an ESI ion source. GC–MS (EI) data were recorded using an Agilent GCMSD 7820A/5977B system. IR spectra were recorded using a Thermo Scientific Nicolet iS10 FTIR (ATR, diamond).

### General Procedure

Amine (1.00 mmol) was dissolved in DMF (5.0 mL) in an 8–10 mL screw cap (silicon/PTFE septum) vial equipped with a small (10 mm × 6 mm) stir bar. The mixture was cooled in an ice–NaCl cooling bath. Then, prepared samples of *O*-(4-nitrobenzoyl)hydroxylamine (274 mg, 1.50 mmol) and diketone (1.10 mmol) were added one by one. The vial was immediately closed, shaken, and placed into a reaction pie block preheated on a stirrer to 85 °C for the reaction time given for a compound. In workup A, the crude mixture was poured into 1 M NaOH (100 mL) and extracted with DCM (3 × 30 mL). The organic phase was washed with brine (2 × 50 mL), dried with anhydrous MgSO_4_, and filtered, and the solvent was evaporated. The product was purified using column chromatography under conditions given for a compound. In workup B, the crude mixture was treated with triethylamine (0.5 mL), and DMF was partially evaporated under a nitrogen flow. The residue was adsorbed on silica gel and purified using column chromatography under conditions given for a compound.

#### 1-(3,3-Dimethylbutan-2-yl)-3,5-dimethyl-1*H*-pyrazole (**1a**)

3,3-Dimethylbutan-2-amine **a** (134 μL, 1.00 mmol), 2,4-pentanedione **1** (114 μL, 1.10 mmol), *O*-(4-nitrobenzoyl)hydroxylamine (273 mg, 1.50 mmol), and DMF (5.0 mL) were used. The reaction was run at 85 °C (reaction block) for 1.5 h. Workup A and chromatography (basic alumina grade I, pentane–Et_2_O 0–60%) were applied to obtain 80 mg (0.44 mmol, 44%) of **1a** as a colorless volatile liquid. For a large-scale experiment, dimethylbutan-2-amine **a** (410 μL, 3.00 mmol), 2,4-pentanedione **1** (342 μL, 3.30 mmol), *O*-(4-nitrobenzoyl)hydroxylamine (820 mg, 4.50 mmol), and DMF (20 mL) were used to obtain 243 mg (1.35 mmol, 45%) of **1a**. ^1^H NMR (500 MHz, CDCl_3_): δ 5.72 (s, 1H), 3.85 (q, *J* = 6.9 Hz, 1H), 2.22 (s, 3H), 2.21 (s, 3H), 1.44 (d, *J* = 6.9 Hz, 3H), 0.93 (s, 9H). ^13^C{^1^H} NMR (126 MHz, CDCl_3_): δ 146.5, 138.9, 104.0, 61.0, 36.4, 27.2, 15.9, 13.9, 12.0. IR (ATR, diamond, cm^–1^): 2955, 2870, 1553, 1456, 1419, 1373, 1365, 1255, 1075, 974, 773. HRMS (ESI) *m*/*z*: [M + H]^+^ calcd for C_11_H_21_N_2_, 181.1700; found, 181.1706.

#### 3,5-Dimethyl-1-(2,4,4-trimethylpentan-2-yl)-1*H*-pyrazole (**1b**)

Compound **1b** was synthesized according to the general procedure using 2,4,4-trimethylpentan-2-amine **b** (161 μL, 1.00 mmol), 2,4-pentanedione **1** (114 μL 1.10 mmol), *O*-(4-nitrobenzoyl)hydroxylamine (273 mg, 1.50 mmol), and DMF (5.0 mL). The reaction was run at 85 °C (reaction block) for 1.5 h. Workup A and chromatography (silica gel, hexane–EA 0–30%) were applied to obtain 79 mg (0.38 mmol, 38%) of **1b** as a yellowish oil. ^1^H NMR (500 MHz, CDCl_3_): δ 5.70 (s, 1H), 2.32 (s, 3H), 2.12 (s, 3H), 1.76 (s, 2H), 1.62 (s, 6H), 0.71 (s, 9H). ^13^C{^1^H} NMR (126 MHz, CDCl_3_): δ 144.6, 138.7, 108.1, 62.5, 53.3, 31.5, 31.0, 30.7, 15.0, 13.5. HRMS (ESI) *m/*z: [M + Na]^+^ calcd for C_13_H_24_N_2_Na, 231.1832; found, 231.1851. IR (ATR, diamond, cm^–1^): 2949, 1550, 1447, 1417, 1357, 1234, 1094, 1024, 776, 603.

#### 3-(2-(3,5-Dimethyl-1*H*-pyrazol-1-yl)ethyl)-1*H*-indole (**1c**)

Tryptamine **c** (160 mg, 1.00 mmol), 2,4-pentanedione **1** (114 μL, 1.10 mmol), *O*-(4-nitrobenzoyl)hydroxylamine (273 mg, 1.50 mmol), and DMF (5.0 mL) were used. The reaction was run at 85 °C (reaction block) for 1.5 h. Workup A and chromatography (basic alumina grade I, hexane–THF 5–100%) were applied to obtain 72 mg (0.30 mmol, 30%) of **1c** as a yellowish solid. ^1^H NMR (500 MHz, CDCl_3_): δ 8.34 (s, 1H), 7.55 (d, *J* = 8.0 Hz, 1H), 7.35 (d, *J* = 8.0 Hz, 1H), 7.23–7.16 (m, 1H), 7.15–7.08 (m, 1H), 6.84 (d, *J* = 2.3 Hz, 1H), 5.74 (s, 1H), 4.23 (t, *J* = 7.4 Hz, 2H), 3.25 (t, *J* = 7.4 Hz, 2H), 2.28 (s, 3H), 1.95 (s, 3H). ^13^C{^1^H} NMR (126 MHz, CDCl_3_): δ 147.5, 139.2, 136.3, 127.4, 122.5, 122.1, 119.5, 118.6, 112.6, 111.3, 104.8, 49.4, 26.7, 13.7, 10.9. IR (ATR, diamond, cm^–1^): 3215, 3184, 2931, 2856, 1551, 1454, 1352, 1232, 1107, 786, 765, 736. HRMS (ESI) *m/*z: [M + H]^+^ calcd for C_15_H_18_N_3_, 240.1496; found, 240.1493.

#### 3,5-Dimethyl-1-(*tert*-pentyl)-1*H*-pyrazole (**1d**)

2-Methylbutan-2-amine **d** (116 μL, 1.00 mmol), 2,4-pentanedione **1** (114 μL 1.10 mmol), *O*-(4-nitrobenzoyl)hydroxylamine (273 mg, 1.50 mmol), and DMF (5.0 mL) were used. The reaction was run at 85 °C (reaction block) for 1.5 h. Workup A and chromatography (silica gel, hexane–EA 30%) were applied to obtain 60 mg (0.361 mmol, 36%) of **1d** as a yellowish volatile liquid. ^1^H NMR (500 MHz, CDCl_3_): δ 5.75 (s, 1H), 2.33 (s, 1H), 2.16 (s, 1H), 1.85 (q, *J* = 7.4 Hz, 1H), 1.56 (s, 2H), 0.71 (t, *J* = 7.4 Hz, 1H). ^13^C{^1^H} NMR (126 MHz, CDCl_3_): δ 145.0, 138.7, 107.7, 62.1, 34.8, 28.0, 14.5, 13.5, 8.4. HRMS (ESI) *m/*z: [M + Na]^+^ calcd for C_10_H_18_N_2_Na, 189.1363; found, 189.1359. IR (ATR, diamond, cm^–1^): 2970, 1550, 1416, 1356, 1304, 1251, 1210, 1107, 1025, 776.

#### 1-Dodecyl-3,5-dimethyl-1*H*-pyrazole (**1e**)

Dodecylamine **e** (185 mg, 1.00 mmol), 2,4-pentanedione **1** (114 μL 1.10 mmol), *O*-(4-nitrobenzoyl)hydroxylamine (273 mg, 1.50 mmol), and DMF (5.0 mL) were used. The reaction was run at 85 °C (reaction block) for 1.5 h. Workup A and chromatography (silica gel, hexane–EA 0–30%) were applied to obtain 87 mg (0.33 mmol, 33%) of **1e** as a yellowish oil. ^1^H NMR (500 MHz, CDCl_3_): δ 5.74 (s, 1H), 3.92–3.88 (m, 2H), 2.19 (s, 6H), 1.78–1.70 (m, 2H), 1.23 (s, 18H), 0.86 (t, *J* = 7.0 Hz, 3H). ^13^C{^1^H} NMR (126 MHz, CDCl_3_): δ 147.0, 138.3, 104.6, 48.7, 31.9, 30.5, 29.6, 29.5, 29.5, 29.3, 29.2, 26.7, 22.6, 14.1, 13.4, 11.0. HRMS (ESI) *m/*z: [M + H]^+^ calcd for C_17_H_33_N_2_, 265.2639; found, 265.2636. IR (ATR, diamond, cm^–1^): 2928, 2854, 1670, 1611, 1553, 1466, 1378, 1310, 1023,774.

#### 1-Cyclohexyl-3,5-dimethyl-1*H*-pyrazole (**1f**).^34^

Cyclohexanamine **f** (115 μL 1.00 mmol), 2,4-pentanedione **1** (114 μL 1.10 mmol), *O*-(4-nitrobenzoyl)hydroxylamine (273 mg, 1.50 mmol), and DMF (5.0 mL) were used. The reaction was run at 85 °C (reaction block) for 1.5 h. Workup A and flash chromatography (silica gel, hexane–THF 0–100%) were applied to obtain 63 mg (0.35 mmol, 35%) of **1f** as a yellowish oil. ^1^H NMR (500 MHz, CDCl_3_): δ 5.74 (s, 1H), 3.92–3.88 (m, 2H), 2.19 (s, 6H), 1.78–1.70 (m, 2H), 1.23 (s, 18H), 0.86 (t, *J* = 7.0 Hz, 3H). ^13^C{^1^H} NMR (126 MHz, CDCl_3_): δ 147.0, 138.3, 104.6, 48.7, 31.9, 30.5, 29.6, 29.5, 29.5, 29.3, 29.2, 26.7, 22.6, 14.1, 13.4, 11.0.

#### 6-(3,5-Dimethyl-1*H*-pyrazol-1-yl)-2-methylheptan-2-ol (**1g**)

6-Amino-2-methylheptan-2-ol **g** (741 mg, 5.00 mmol), 2,4-pentanedione **1** (568 μL, 5.48 mmol), *O*-(4-nitrobenzoyl)hydroxylamine (1.37 mg, 7.52 mmol), and DMF (50 mL) were used. The reaction was run at 80 °C (reaction block) for 2 h. Workup A and chromatography (basic alumina grade I, hexane–THF 0–80%) were applied to obtain 383 mg (1.71 mmol, 34%) of **1g** as a yellowish oil. ^1^H NMR (500 MHz, CDCl_3_): δ 5.71 (s, 1H), 4.15–4.03 (m, 1H), 2.20 (s, 3H), 2.19 (s, 3H), 2.05–1.93 (m, 1H), 1.74–1.63 (m, 1H), 1.51 (s, 1H), 1.40 (m, 2H overlap with d, *J* = 6.7 Hz, 3H), 1.31–1.24 (m, 1H), 1.19–1.14 (m, 1H), 1.13 (s, 6H). ^13^C{^1^H} NMR (126 MHz, CDCl_3_): δ 147.2, 138.2, 104.5, 70.9, 53.7, 43.4, 37.1, 29.4, 29.3, 21.3, 21.3, 13.8, 11.2. IR (ATR, diamond, cm^–1^): 3392 (OH), 2969, 2932, 2868, 1552, 1454, 1423, 1375, 1158, 773. HRMS (ESI) *m/*z: [M + H]^+^ calcd for C_13_H_25_N_2_O, 225.1962; found, 225.1959.

#### 1-Adamantan-1-yl-3,5-dimethyl-1*H*-pyrazole (**1h**).^15^

1-Adamantylamine **h** (151,25 mg, 1.00 mmol), 2,4-pentanedione **1** (114 μL 1.10 mmol), *O*-(4-nitrobenzoyl)hydroxylamine (273 mg, 1.50 mmol), and DMF (5.0 mL) were used. The reaction was run at 85 °C (reaction block) for 1.5 h. Workup A and chromatography (silica gel, hexane–EA 30%) were applied to obtain 97 mg (0.42 mmol, 42%) of **1h** as a yellowish oil. ^1^H NMR (500 MHz, CDCl_3_): δ 5.78 (s, 1H), 2.43 (s, 3H), 2.27 (d, *J* = 3.1 Hz, 6H), 2.20 (s, 6H), 1.74 (s, 6H). ^13^C{^1^H} NMR (126 MHz, CDCl_3_): δ 145.3, 138.6, 108.0, 60.4, 42.3, 36.3, 30.0, 15.1, 13.6.

#### 3,5-Dimethyl-1-(1-phenylethyl)-1*H*-pyrazole (**1i**).^15^

1-Phenylethan-1-amine **i** (127 μL, 1.00 mmol), 2,4-pentanedione **1** (114 μL 1.10 mmol), *O*-(4-nitrobenzoyl)hydroxylamine (273 mg, 1.50 mmol), and DMF (5.0 mL) were used. The reaction was run at 80 °C (reaction block) for 1.5 h. Workup A and chromatography (silicagel hexane–EA 30%) were applied to obtain 76 mg (0.38 mmol, 38%) of **1i** as a yellowish oil. ^1^H NMR (500 MHz, CDCl_3_): δ 7.28–7.12 (m, 1H), 7.05–7.00 (m, 1H), 5.76 (s, 1H), 5.27 (q, *J* = 7.1 Hz, 1H), 2.21 (s, 1H), 2.02 (s, 1H), 1.84 (d, *J* = 7.1 Hz, 1H). ^13^C{^1^H} NMR (126 MHz, CDCl_3_): δ 146.9, 143.0, 138.9, 128.5, 127.1, 125.9, 105.5, 57.3, 21.7, 13.7, 11.1.

#### Ethyl 2-(3,5-Dimethyl-1*H*-pyrazol-1-yl)-3-phenylpropanoate (**1j**)

Ethyl phenylalaninate^35^**j** (193 mg, 1.00 mmol), 2,4-pentanedione **1** (114 μL, 1.10 mmol), *O*-(4-nitrobenzoyl)hydroxylamine (273 mg, 1.50 mmol), and DMF (5.0 mL) were used. The reaction was run at 85 °C (reaction block) for 1.5 h. Workup B and chromatography (neutral alumina grade I, hexane–THF 0–40%) were applied to obtain 73 mg (0.27 mmol, 27%) of **1j** as a colorless oil. ^1^H NMR (500 MHz, CDCl_3_): δ 7.22–7.15 (m, 3H), 7.01–6.96 (m, 2H), 5.69 (s, 1H), 4.76 (dd, *J* = 9.8, 5.2 Hz, 1H), 4.20 (qd, *J* = 7.1, 2.2 Hz, 2H), 3.58–3.47 (m, 2H), 2.25 (s, 3H), 1.83 (s, 3H), 1.21 (t, *J* = 7.1 Hz, 3H). ^13^C{^1^H} NMR (75 MHz, CDCl_3_): δ 169.4, 148.4, 140.3, 137.6, 129.2, 128.5, 126.8, 105.1, 62.0, 61.8, 37.4, 14.2, 13.9, 10.7. IR (ATR, diamond, cm^–1^): 2980, 2925, 1745 (CO), 1557, 1455, 1262, 1210, 1173, 1028, 752, 701. HRMS (ESI) *m/*z: [M + H]^+^ calcd for C_16_H_21_N_2_O_2_, 273.1598; found, 273.1597.

#### 1-(Bicyclo[2.2.1]heptan-2-yl)-3,5-dimethyl-1*H*-pyrazole (**1k**)

Bicyclo[2.2.1]heptan-2-amine **k** (60 μL, 0.50 mmol), pentane-2,4-dione **1** (56 μL 0.55 mmol), *O*-(4-nitrobenzoyl)hydroxylamine (136 mg, 0.75 mmol), and DMF (2.5 mL) were used. The reaction was run at 85 °C for 1.5 h. Workup A and chromatography (silica gel, hexane–THF 0–30%) were applied to obtain 33 mg (0.18 mmol, 35%) of **1k** as a yellowish oil. ^1^H NMR (500 MHz, CDCl_3_): δ 5.79 (s, 1H), 3.97–3.96 (m, 1H), 2.42 (s, 1H), 2.37–2.29 (m, 1H), 2.23 (s, 1H), 2.21 (s, 1H), 2.08–2.02 (m, 1H), 1.73–1.70 (m, 1H), 1.63–1.50 (m, 1H), 1.25–1.11 (m, 2H). ^13^C{^1^H} NMR (126 MHz, CDCl_3_): δ 146.2, 138.4, 105.2, 60.6, 43.5, 37.7, 36.0, 35.8, 28.8, 27.7, 13.9, 11.5. IR (ATR, diamond, cm^–1^): 2951, 2923, 1553, 1452, 1378, 1290, 1258, 1023, 802, 773. HRMS (ESI) *m*/*z*: [M + H]^+^ calcd for C_12_H_19_N_2_, 191.1543; found, 191.1549.

#### 3,5-Dimethyl-1-phenyl-1*H*-pyrazole (**1l**).^18^

Aniline **l** (94 μL, 1.0 mmol), 2,4-pentanedione **1** (114 μL 1.10 mmol), *O*-(4-nitrobenzoyl)hydroxylamine (273 mg, 1.50 mmol), and DMF (5.0 mL) were used. The reaction was run at 85 °C (reaction block) for 1.5 h. Workup A and chromatography (silica gel, hexane–EA 30%) were applied to obtain 68 mg (0.47 mmol, 47%) of **1l** as a yellowish oil. ^1^H NMR (500 MHz, CDCl_3_): δ 7.34 (d, *J* = 4.4 Hz, 4H), 7.24 (dq, *J* = 8.7, 4.4 Hz, 1H), 5.91 (s, 1H), 2.22 (d, *J* = 10.9 Hz, 6H). ^13^C{^1^H} NMR (126 MHz, CDCl_3_): δ 148.9, 139.9, 139.3, 128.9, 127.2, 124.7, 106.9, 13.5, 12.3.

#### 1-(4-Methoxyphenyl)-3,5-dimethyl-1*H*-pyrazole (**1m**).^18^

4-Methoxyaniline **m** (123 mg, 1.00 mmol), 2,4-pentanedione **1** (114 μL 1.10 mmol), *O*-(4-nitrobenzoyl)hydroxylamine (273 mg, 1.50 mmol), and DMF (5.0 mL) were used. The reaction was run at 85 °C (reaction block) for 1.5 h. Workup A and chromatography (silica gel, hexane–EA 30%) were applied to obtain 112 mg (0.55 mmol, 55%) of **1m** as a brown oil. ^1^H NMR (500 MHz, CDCl_3_): δ 7.33–7.27 (m, 2H), 6.96–6.90 (m, 2H), 5.94 (s, 3H), 2.27 (s, 3H), 2.22 (s, 3H). ^13^C{^1^H} NMR (126 MHz, CDCl_3_): δ 158.7, 148.5, 139.4, 133.1, 126.4, 114.1, 106.2, 55.5, 13.5, 12.1.

#### 1-(4-Fluorophenyl)-3,5-dimethyl-1*H*-pyrazole (**1n**).^36^

4-Fluoroaniline **n** (95 μL, 1.0 mmol), 2,4-pentanedione **1** (114 μL 1.10 mmol), *O*-(4-nitrobenzoyl)hydroxylamine (273 mg, 1.50 mmol), and DMF (5.0 mL) were used. The reaction was run at 85 °C (reaction block) for 1.5 h. Workup A and chromatography (silica gel, hexane–EA 30%) were applied to obtain 115 mg (0.60 mmol, 60%) of **1n** as a yellowish oil. ^1^H NMR (500 MHz, CDCl_3_): δ 7.39–7.33 (m, 2H), 7.15–7.06 (m, 2H), 5.96 (s, 1H), 2.25 (d, *J* = 10.3 Hz, 6H). ^13^C{^1^H} NMR (126 MHz, CDCl_3_): δ 161.6 (d, *J* = 247.0 Hz), 149.1, 139.5, 136.1 (d, *J* = 3.0 Hz), 126.6 (d, *J* = 8.6 Hz), 115.9 (d, *J* = 22.8 Hz), 106.9, 13.5, 12.2.

#### 3,5-Dimethyl-1-(naphthalen-2-yl)-1*H*-pyrazole (**1o**)

Naphthalen-2-amine **o** (143 mg, 1.00 mmol), 2,4-pentanedione **1** (114 μL 1.10 mmol), *O*-(4-nitrobenzoyl)hydroxylamine (273 mg, 1.50 mmol), and DMF (5.0 mL) were used. The reaction was run at 85 °C (reaction block) for 1.5 h. Workup A and chromatography (silica gel, hexane–EA 0–30%) were applied to obtain 155 mg (0.70 mmol, 70%) of **1o** as a red oil. ^1^H NMR (500 MHz, CDCl_3_): δ 7.90 (d, *J* = 8.7 Hz, 1H), 7.88–7.82 (m, 3H), 7.59 (dd, *J* = 8.7, 2.1 Hz, 1H), 7.54–7.43 (m, 2H), 6.02 (s, 1H), 2.35 (s, 3H), 2.32 (s, 3H). ^13^C{^1^H} NMR (126 MHz, CDCl_3_): δ 148.5, 133.1, 132.7, 129.5, 128.2, 127.9, 127.1, 126.9, 123.6, 123.1, 107.5, 12.9, 12.4. HRMS (ESI) *m/*z: [M + Na]^+^ calcd for C_15_H_14_N_2_Na 245.1050; found, 245.1045. IR (ATR, diamond, cm^–1^): 3053, 2920, 2852, 1633, 1508, 1379, 1266, 857, 815, 783, 472.

#### 1-(5-Bromo-2-methylphenyl)-3,5-dimethyl-1*H*-pyrazole (**1p**)

5-Bromo-2-methylaniline **p** (191 mg, 1.00 mmol), 2,4-pentanedione **1** (114 μL, 1.10 mmol), *O*-(4-nitrobenzoyl)hydroxylamine (273 mg, 1.50 mmol), and DMF (5.0 mL) were used. The reaction was run at 80 °C (reaction block) for 16 h. Workup A and chromatography (basic alumina grade I, hexane–THF 0–30%) were applied to obtain 149 mg (0.56 mmol, 56%) of **1p** as a yellowish oil. ^1^H NMR (500 MHz, CDCl_3_): δ 7.45 (dd, *J* = 8.2, 2.1 Hz, 1H), 7.39 (d, *J* = 2.1 Hz, 1H), 7.17 (d, *J* = 8.2 Hz, 1H), 5.96 (s, 1H), 2.27 (s, 3H), 2.06 (s, 3H), 2.01 (s, 3H). ^13^C{^1^H} NMR (126 MHz, CDCl_3_): δ 149.2, 140.4, 140.0, 135.6, 132.2, 132.1, 131.1, 119.2, 105.5, 17.0, 13.7, 11.4. IR (ATR, diamond, cm^–1^): 2954, 2924, 2857, 1594, 1556, 1496, 1424, 1360, 1036, 823, 782. HRMS (ESI) *m/*z: [M + H]^+^ calcd for C_12_H_14_N_2_Br, 265.0335; found, 265.0335.

#### 3,4,5-Ttrimethyl-1-(2,4,4-trimethylpentan-2-yl)-1*H*-pyrazole (**2b**)

2,4,4-Trimethylpentan-2-amine **b** (161 μL, 1.00 mmol), 3-methylpentane-2,4-dione **2** (128 μL 1.10 mmol), *O*-(4-nitrobenzoyl)hydroxylamine (273 mg, 1.50 mmol), and DMF (5 mL) were used. The reaction was run at 85 °C (reaction block) for 1.5 h. Workup A and chromatography (silica gel, hexane–EA 0–30%) were applied to obtain 95 mg (0.43 mmol, 43%) of **2b** as a yellowish oil. ^1^H NMR (500 MHz, CDCl_3_): δ 2.30 (s, 1H), 2.21–2.08 (m, 1H), 1.86 (s, 1H), 1.82 (s, 1H), 1.68 (s, 2H), 0.77 (s, 3H). ^13^C{^1^H} NMR (126 MHz, CDCl_3_): δ 143.3, 135.2, 113.1, 62.3, 53.2, 31.5, 30.7, 29.5, 13.1, 11.8, 8.0. IR (ATR, diamond, cm^–1^): 2954, 2252, 2669, 1669, 1568, 1540, 1415, 1050, 905, 729 HRMS (ESI) *m/*z: [M + Na]^+^ calcd for C_14_H_26_N_2_Na, 245.1989; found, 245.1987.

#### 3,5-Diethyl-1-(2,4,4-trimethylpentan-2-yl)-1*H*-pyrazole (**3b**)

2,4,4-Trimethylpentan-2-amine **b** (161 μL, 1.00 mmol), heptane-3,5-dione **3** (150 μL 1.1 mmol), *O*-(4-nitrobenzoyl)hydroxylamine (273 mg, 1.50 mmol), and DMF (5 mL) were used. The reaction was run at 85 °C (reaction block) for 1.5 h. Workup A and chromatography (silica gel, hexane–EA 0–30%) were applied to obtain 88 mg (0.37 mmol, 37%) of **3b** as a yellowish oil. ^1^H NMR (500 MHz, CDCl_3_): δ 5.87 (s, 1H), 2.77 (q, *J* = 7.4 Hz, 2H), 2.56 (q, *J* = 7.6 Hz, 2H), 1.80 (s, 2H), 1.68 (s, 7H), 1.26 (t, *J* = 7.4 Hz, 3H), 1.18 (t, *J* = 7.6 Hz, 3H), 0.73 (s, 9H). ^13^C{^1^H} NMR (126 MHz, CDCl_3_): δ 150.8, 145.5, 103.6, 62.6, 53.5, 31.6, 31.3, 30.7, 21.9, 21.6, 14.4, 13.6. HRMS (ESI) *m/*z: [M + H]^+^ calcd for C_15_H_29_N_2_ 237.2326; found, 237.2323. IR (ATR, diamond, cm^–1^): 3001, 2929, 1545, 1465, 1365, 1253 1227, 1089, 956, 773.

#### 4-Ethyl-3,5-dimethyl-1-(2,4,4-trimethylpentan-2-yl)-1*H*-pyrazole (**4b**)

2,4,4-Trimethylpentan-2-amine **b** (67 mg, 0.50 mmol), 3-ethylpentane-2,4-dione **4** (336 μL, 2.50 mmol), *O*-(4-nitrobenzoyl)hydroxylamine (136 mg, 0.75 mmol), and DMF (2.5 mL) were used. The reaction was run at 85 °C (reaction block) for 1.5 h. Workup A and chromatography (basic alumina grade I, hexane-(DCM-MeOH 10%) 0–20%) were applied to obtain 28 mg (0.118 mmol, 26%) of **4b** as a colorless oil. ^1^H NMR (500 MHz, CDCl_3_): δ 2.33–2.28 (m, 5H), 2.16 (s, 3H), 1.82 (s, 2H), 1.69 (s, 6H), 1.00 (t, *J* = 7.6 Hz, 3H), 0.75 (s, 9H). ^13^C{^1^H} NMR (126 MHz, CDCl3): δ 142.9, 135.1, 120.4, 62.4, 53.4, 31.7, 31.2, 30.9, 17.0, 15.6, 13.0, 12.0. IR (ATR, diamond, cm^–1^): 2956, 2925, 2855, 1463, 1366, 1355, 1272, 1256, 1235. HRMS (ESI) *m*/*z*: [M + H]^+^ calcd for C_15_H_29_N_2_, 237.2326; found, 237.2326.

#### 3-Methyl-5-phenyl-1-(2,4,4-trimethylpentan-2-yl)-1*H*-pyrazole (**5b**)

2,4,4-Trimethylpentan-2-amine **b** (26 mg, 0.20 mmol), 1-phenylbutane-1,3-dione **5** (162 mg, 1.0 mmol), *O*-(4-nitrobenzoyl)hydroxylamine (55 mg, 0.30 mmol), and DMF (1 mL) were used. The reaction was run at 85 °C (reaction block) for 1.5 h. Workup A and chromatography (silica gel, hexane–EA 0–30%) were applied to obtain 25 mg (0.092 mmol, 46%) of **5b** as a white solid. ^1^H NMR (500 MHz, CDCl_3_): δ 7.67–7.26 (m, 5H), 5.88 (s, 1H), 2.26 (s, 3H), 1.78 (s, 2H), 1.46 (s, 6H), 0.79 (s, 9H). ^13^C{^1^H} NMR (126 MHz, CDCl_3_): δ 144.6, 143.3, 135.0, 130.5, 128.1, 127.6, 109.3, 64.1, 54.8, 31.8, 31.5, 30.7, 13.5, 13.5. IR (ATR, diamond, cm^–1^): 2955, 1545, 1498, 1442, 1395, 1365, 1253, 1196, 793, 704. HRMS (ESI) *m/*z: [M + Na]^+^ calcd for C_18_H_26_N_2_Na, 293.1989; found, 293.1989.

#### 5-Isobutyl-3-methyl-1-(2,4,4-trimethylpentan-2-yl)-1*H*-pyrazole (**6b**)

2,4,4-Trimethylpentan-2-amine **b** (161 μL, 1.00 mmol), 6-methylheptane-2,4-dione **6** (173 μL, 1.10 mmol), *O*-(4-nitrobenzoyl)hydroxylamine (273 mg, 1.50 mmol), and DMF (5.0 mL) were used. The reaction was run at 85 °C (reaction block) for 2 h. Workup A and chromatography (basic alumina grade I, hexane–THF 0–20%) were applied to obtain 97 mg (0.37 mmol, 37%) of **6b** as a yellowish oil. ^1^H NMR (500 MHz, CDCl_3_): δ 5.84 (s, 1H), 2.61 (d, *J* = 7.1 Hz, 2H), 2.20 (s, 3H), 2.03–1.94 (m, 1H), 1.83 (s, 2H), 1.69 (s, 6H), 1.01 (d, *J* = 6.6 Hz, 6H), 0.77 (s, 9H). ^13^C{^1^H} NMR (126 MHz, CDCl_3_): δ 144.6, 143.3, 106.6, 62.8, 53.8, 37.9, 31.7, 31.5, 30.9, 28.5, 23.1, 13.8. IR (ATR, diamond, cm^–1^): 2954, 2870, 1546, 1468, 1425, 1387, 1363, 1241, 1021, 777, 600. HRMS (ESI) *m/*z: [M + H]^+^ calcd for C_16_H_31_N_2_, 251.2482; found, 251.2481.

## Supporting Information Available

Copies of ^1^H and ^13^C spectra of products, X-ray data, additional experiments details, and full optimization table (PDF)

## Accession Codes

CCDC 2019272 (**1n**), and 2019718 (**4b**) contains the supplementary crystallographic data for this paper. These data can be obtained free of charge via www.ccdc.cam.ac.uk/data_request/cif, or by emailing data_request@ccdc.cam.ac.uk, or by contacting The Cambridge Crystallographic Data Centre, 12 Union Road, Cambridge CB2 1EZ, UK; fax: + 44 1223 336033.
