# Supplementary material for: Direct Preparation of N-Substituted Pyrazoles from Primary Aliphatic or Aromatic Amines
Source: J Org Chem. 2021 Jul 1;86(14):9353–9. doi: 10.1021/acs.joc.1c00606 (PMC8389898; doi:10.1021/acs.joc.1c00606)

Supporting Information

Direct preparation of *N*-substituted pyrazoles from primary aliphatic or aromatic amines.

Nurbey Gulia,* Marcin Małecki, Sławomir Szafert

Faculty of Chemistry, University of Wrocław, 14 F. Joliot-Curie, 50-383 Wrocław, Poland
e-mail: nurbey.gulia@chem.uni.wroc.pl, http://zb1.chem.uni.wroc.

Contents

1. USED CHEMICALS S2
2. ADDITIONAL EXPERIMENTS S2
3. REACTION CONDITIONS OPTIMIZATION. S4
4. CRYSTALLOGRAPHY S5
5. NMR SPECTRA S7

1. Used chemicals

Further chemicals were obtained from commercial sources and used without purification: *O*-(4-nitrobenzoyl)hydroxylamine (cas: 35657-36-4, ABCR), hydroxylamine-*O*-sulfonic acid (cas: 2950-43-8, Alfa Aesar, 97%), *O*-(2,4-dinitrophenyl)hydroxylamine (cas: 17508-17-7, Carbosynth), *O*-(diphenylphosphinyl)hydroxylamine (cas: 72804-96-7, Carbosynth), ethyl *O*-(2-mesitylenesulfonyl)acethydroxamate (cas: 38202-27-6, Carbosynth), anhydrous $MgSO_4$ (Chempur, puriss p.a.), acetic acid (Honeywell, ACS reagent), sodium acetate (Aldrich, ACS reagent), K_2CO_3 (Aldrich, ACS reagent), *N*,*N*-diisopropylethylamine (cas: 7087-68-5, Acros, 98%), NaOH (Chempur, puriss p.a.), 3,3-dimethylbutan-2-amine (cas: 3850-30-4, Alfa Aesar, 98%), 2,4,4-trimethylpentan-2-amine (cas: 107-45-9, Aldrich, 95%), tryptamine (cas: 61-54-1, no name, structure confirmed by ^{1}HNMR), acetylacetone (cas: 123-54-6, Fluka, 99%), 2-methylbutan-2-amine (cas: 594-39-8, Alfa Aesar, 98%), dodecylamine (cas: 124-22-1, Aldrich, 99%), cyclohexanamine (cas: 108-91-8, Aldrich, 98%), amino-2-methylheptan-2-ol (cas: 372-66-7, Acros, 98%), 1-adamantylamine (cas: 768-94-5, Aldrich, 98%), 1-phenylethan-1-amine (cas: 618-36-0, Acros, 98%), phenylalanine (cas: 63-91-2, Reanal, 98%), bicyclo[2.2.1]heptan-2-amine (cas: 7242-92-4, Aldrich, 99%), aniline (cas: 62-53-3, Chempur, 99%), 4-methoxyaniline (cas: 104-94-9, Acros, 98%), 4-fluoroaniline (cas: 371-40-4, Acros, 98%), naphthalen-2-amine (cas: 91-59-8, POCH) 5-bromo-2-methylaniline (cas:39478-78-9, Aldrich, 97%), 6-methylheptane-2,4-dione (cas:3002-23-1, Aldrich, 98%), heptane-3,5-dione (cas: 7424-54-6, Alfa Aesar, 98%), 3-ethylpentane-2,4-dione (cas:1540-34-7, Aldrich, 98%), 1-phenylbutane-1,3-dione (cas: 93-91-4, Aldrich, 99%), 6-methylheptane-2,4-dione (cas:3002-23-1, Aldrich, 98%). Silica gel 60 Å 0.06-0.20 mm (Merck); Silica gel 60 Å 0.04-0.06 mm (Merck); Al_2O_3, basic alumina, Brockmann grade I, 60 mesh (Alfa Aesar); Al_2O_3, neutral alumina, Brockmann grade I, 60 mesh (Alfa Aesar); TLC plates, silica gel 60 Å (Macherey Nagel) were used for products purifications.

2. Additional experiments

*Reaction of amine **p** with amination reagent **R1** without diketone.*

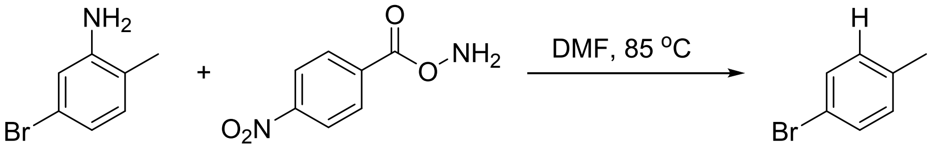

The reaction was conducted under the standard condition on 0.1 mmol scale without diketone using 3.0 equivalents **R1**. The crude mixture was analyzed by GC-MS.

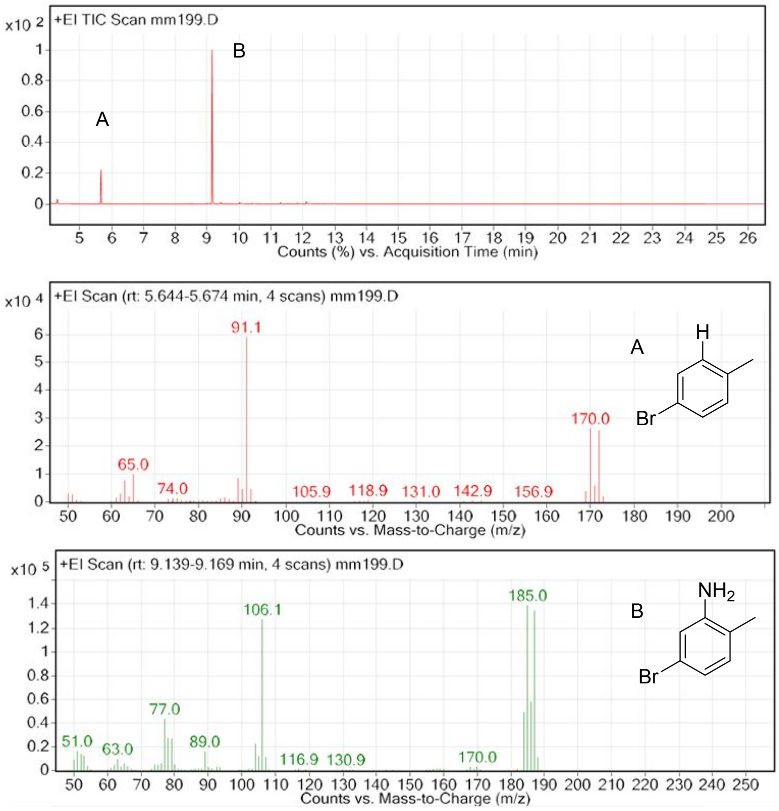

*GC-MS of crude reaction mixture of **1a**.*

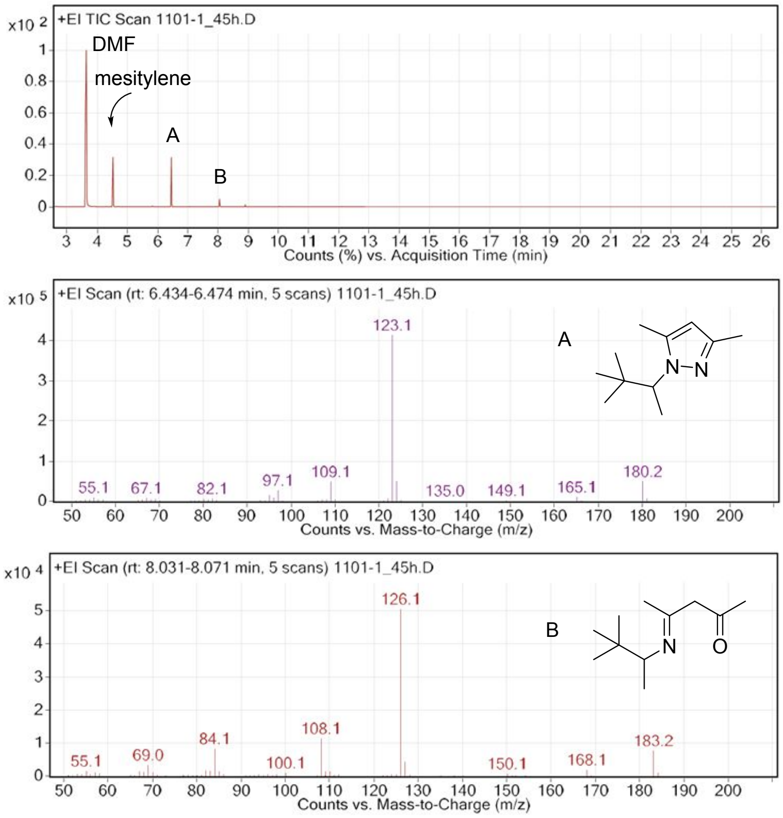

3. Reaction conditions optimization.

Table S1. The full table for reaction conditions and amination reagent optimization.

[a]	**2** [equiv]	**3** [equiv]	Additive [equiv]	Order	Solvent [M]	Temp. [ºC]	Time [h]	Yield[b] [%]
1[c]	1.0	**R4 [1.0]**		a+1/+R	MeCN [1.0]	70-130	16	-
2[c]	1.0	**R2 [1.0]**		a+1/+R	MeCN [1.0]	70-130	16	-
3[c]	1.0	**R1 [1.0]**		a+1/+R	MeCN [1.0]	70-130	16	14
4	1.0	R1 [1.0]		**a+1+R**	MeCN [1.0]	**RT-130**	16	29
5	1.1	R1 [1.5]		a+1+R	**MeCN [0.2]**	RT-110	2	38
6	1.1	R1 [1.5]		a+1+R	**DMF [0.2]**	RT-110	2	49
7	1.1	R1 [1.5]		a+1+R	**EtOH [0.2]**	RT-110	2	33
8	1.1	R1 [1.5]		a+1+R	**Toluene [0.2]**	RT-110	2	34
9[d]	1.1	**R1 [1.5+1.0]**		a+1+R	MeCN [0.2]	RT-110	2+2	31
10	1.1	R1 [1.5]		a+1+R	DMF [0.2]	**0-110**	1.5	54
11	1.1	R1 [1.5]		a+1+R	DMF [0.2]	**0-95**	1.5	55
12	1.1	R1 [1.5]		a+1+R	DMF [0.2]	**0-85**	1.5	54
13	1.1	R1 [1.5]		a+1+R	DMF [0.2]	**0-50**	1.5	41
14	1.1	R1 [1.5]		a+1+R	DMF [0.2]	**0-RT**	1.5	31
15	1.1	R1 [1.5]		a+1+R	DMF [0.2]	**0-RT**	**24**	34
16	1.1	R1 [1.5]		a+1+R	**DMSO [0.2]**	0-85	1.5	31
17	1.1	**R1 [1.1]**		a+1+R	DMF [0.2]	0-85	1.5	47
18	1.1	**R1 [1.3]**		a+1+R	DMF [0.2]	0-85	1.5	50
19	1.1	**R1 [1.7]**		a+1+R	DMF [0.2]	0-85	1.5	48
20	1.1	**R1 [1.9]**		a+1+R	DMF [0.2]	0-85	1.5	48
21[e]	1.1	R1 [1.5]		**a+R/+1**	DMF [0.2]	0-85	1.5	17
22	1.1	R1 [1.5]		**a+R+1**	DMF [0.2]	0-85	1.5	52
23	1.1	R1 [1.5]		**1+R+a**	DMF [0.2]	0-85	1.5	54
24	**1.1**	R1 [1.5]		**a+1+R**	DMF [0.2]	0-85	1.5	54
25	**1.0**	R1 [1.5]		a+1+R	DMF [0.2]	0-85	1.5	51
26	**1.3**	R1 [1.5]		a+1+R	DMF [0.2]	0-85	1.5	51
27	**1.5**	R1 [1.5]		a+1+R	DMF [0.2]	0-85	1.5	48
28	**1.7**	R1 [1.5]		a+1+R	DMF [0.2]	0-85	1.5	47
29	1.1	R1 [1.5]	**H_2O [1.0]**	a+1+R	DMF [0.2]	0-85	1.5	51
30	1.1	R1 [1.5]	**H_2O [2.7]**	a+1+R	DMF [0.2]	0-85	1.5	43
31	1.1	R1 [1.5]	**$MgSO_4$ [2.5]**	a+1+R	DMF [0.2]	0-85	14	42
32	1.1	**R1 [1.5]**		a+1+R	DMF [0.2]	0-85	1.5	53[f]
33	1.1	**R2 [1.5]**		a+1+R	DMF [0.2]	0-85	1.5	23[f]
34	1.1	**R3 [1.5]**		a+1+R	DMF [0.2]	0-85	1.5	41[f]
35	1.1	**R4 [1.5]**		a+1+R	DMF [0.2]	0-85	1.5	0[f]
36	1.1	**R5 [1.5]**		a+1+R	DMF [0.2]	0-85	1.5	0[f]
37	1.1	**R6 [1.5]**		a+1+R	DMF [0.2]	0-85	1.5	27[f]
38	1.1	**R6 [1.5]**[g]		a+1+R	DMF [0.2]	0-85	1.5	53[f]
39	1.1	R1 [1.5]	**AcOH [1.0]**	a+1+R	DMF [0.2]	0-85	1.5	51[f]
40	1.1	R1 [1.5]	**AcONa [1.0]**	a+1+R	DMF [0.2]	0-85	1.5	53[f]
41	1.1	R1 [1.5]	**K_2CO_3 [1.0]**	a+1+R	DMF [0.2]	0-85	1.5	56[f]
42	1.1	R1 [1.5]	**DIPEA [1.0]**	a+1+R	DMF [0.2]	0-85	1.5	46[f]

[a] Reactions were carried out using **a** (0.2 mmol, 1.0 equiv), **1** (1.1-1.5 equiv), **R1-5** (1.1-1.7 equiv), DMF (1.0 mL, 0.2 M), at given temperature under air.; [b] GC yield; [c] Before **R** was added the mixture of **a** and **1** was heated for 1.5 h.; [d] After 2 h at 110 ºC, the next portion of **R** was added.; [e] **1** was added after the mixture heated to 80 ºC; [f] NMR yield.; [g] **R6** was prepared *in situ*.; [h] wet **R6** used.

4. Crystallography

To obtain crystals of 3,5-dimethyl-1-(naphthalen-2-yl)-1*H*-pyrazole **1o** appropriate for x-ray structure determination, a sample of **1o** (10 mg) was dissolved in $CHCl_3$ (0.6 mL) in an NMR tube and close with a standard plastic cap. The solvent was slow evaporated for two weeks at room temperature to obtain colorless plates of **1o**.

To obtain crystals of 3-methyl-5-phenyl-1-(2,4,4-trimethylpentan-2-yl)-1*H*-pyrazole **5b** a sample of **5b** (10 mg) was dissolved in $CHCl_3$ (0.6 mL) in 2.0 mL screw cap silicon septa vial. The solvent was slow evaporated for two weeks at room temperature to obtain colorless blocks of **5b**.

X-ray diffraction data were collected with a KUMA KM4 CCD diffractometer (ω scan technique). The space groups were determined from systematic absences and Lorentz and polarization corrections were applied. Absorption correction was applied with the use of CrysAlisPro software.[1] The structures were solved by direct methods and refined by full-matrix, least-squares on F2 by use of the Olex.[2] Non-hydrogen atoms were refined with anisotropic thermal parameters. Hydrogen atoms positions were calculated and added to the structure factor calculations but were not refined.

Figure S1. ORTEP view of the molecular structure of **1o** (2019718) and **5b** (2019272). Thermal ellipsoids are drawn to encompass 50 % probability. Hydrogen atoms are omitted for clarity.

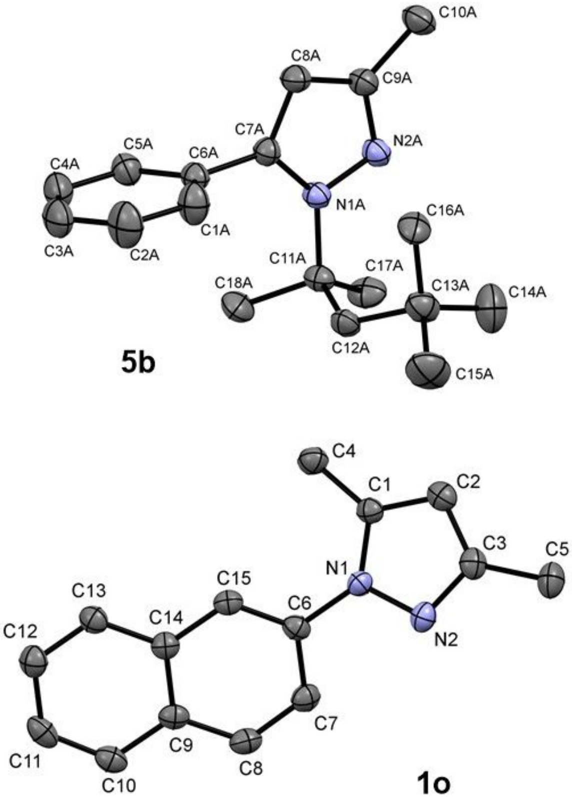

Table S1. X-ray experimental data and refinement.

	5b	***1o***
Crystallographic data accession code	2019718	2019272
Empirical formula	$C_{36}H_{52}N_4$	$C_{15}H_{14}N_2$
Formula weight	540.81	222.28
Crystal system	Monoclinic	Orthorhombic
Space group	$P2_1/c$	$Pca2_1$
a (Å)	27.0381(7)	23.6984(15)
b (Å)	8.1714(2)	6.7532(4)
c (Å)	15.4541(4)	7.4343(4)
α (°)	90.0	90.0
β (°)	106.255(3)	90.0
γ (°)	90.0	90.0
V (Å^3)	3277.92(15)	1189.79(12)
Z	4	4
Crystal description	block, colourless	plate, colourless
Crystal size (mm)	0.784 × 0.499 × 0.385	0.583 × 0.344 × 0.023
d_{calc} (g/cm3)	1.093	1.241
μ (mm-1)	0.064	0.074
F(000)	1181.0	472.0
λ (Å)	0.71073	0.71073
T (K)	100	100
Θ min/max (°)	3.1/28.7	6.032/ 73.124
h, k, l min/max	-35 / 45, -10 / 12, -26 /19	-30 /h ≤ 30, -8 /k ≤ 6, -12 / 9
Reflections collected	43064	7930
Independent reflections	13185	2758
Reflections with I>2σ(I)	13185	2758
No. of parameters	503	184
R (int.)	0.0704	0.0729
R indices ($F^2 > 2\sigma(F^2)$)	0.056	0.0661
R indices (wR(F^2))	0.162	0.1542
GooF	1.032	1.042
Δρmax/Δρmin (e·Å^{-3})	0.27 /-0.36	0.25 /-0.21

(1) *CrysAlisPRO*; Oxford Diffraction /Agilent Technologies UK Ltd: Yarnton, England.

(2) Dolomanov, O. V.; Bourhis, L. J.; Gildea, R. J.; Howard, J. A. K.; Puschmann, H. *OLEX2* : A Complete Structure Solution, Refinement and Analysis Program. *J. Appl. Crystallogr.* **2009**, *42* (2), 339–341. https://doi.org/10.1107/S0021889808042726.

5. NMR spectra

1-(3,3-Dimethylbutan-2-yl)-3,5-dimethyl-1*H*-pyrazole, **1a**.

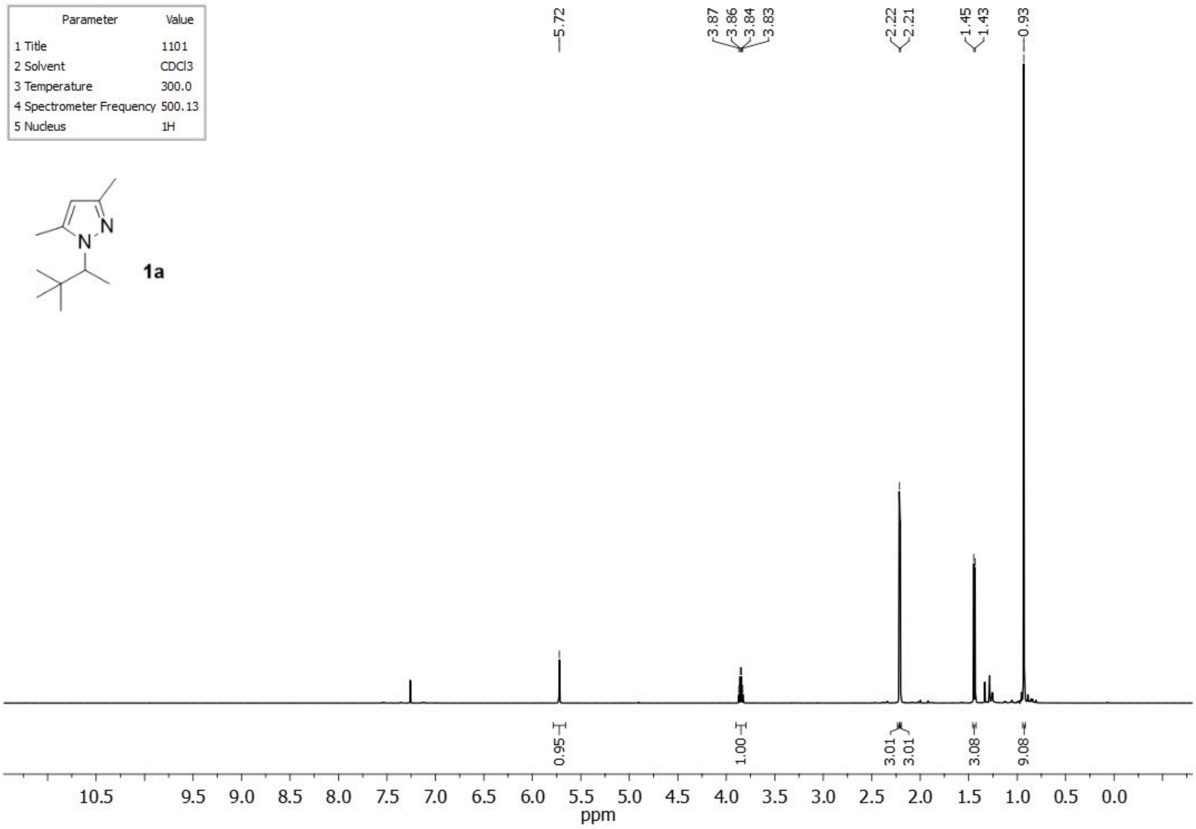

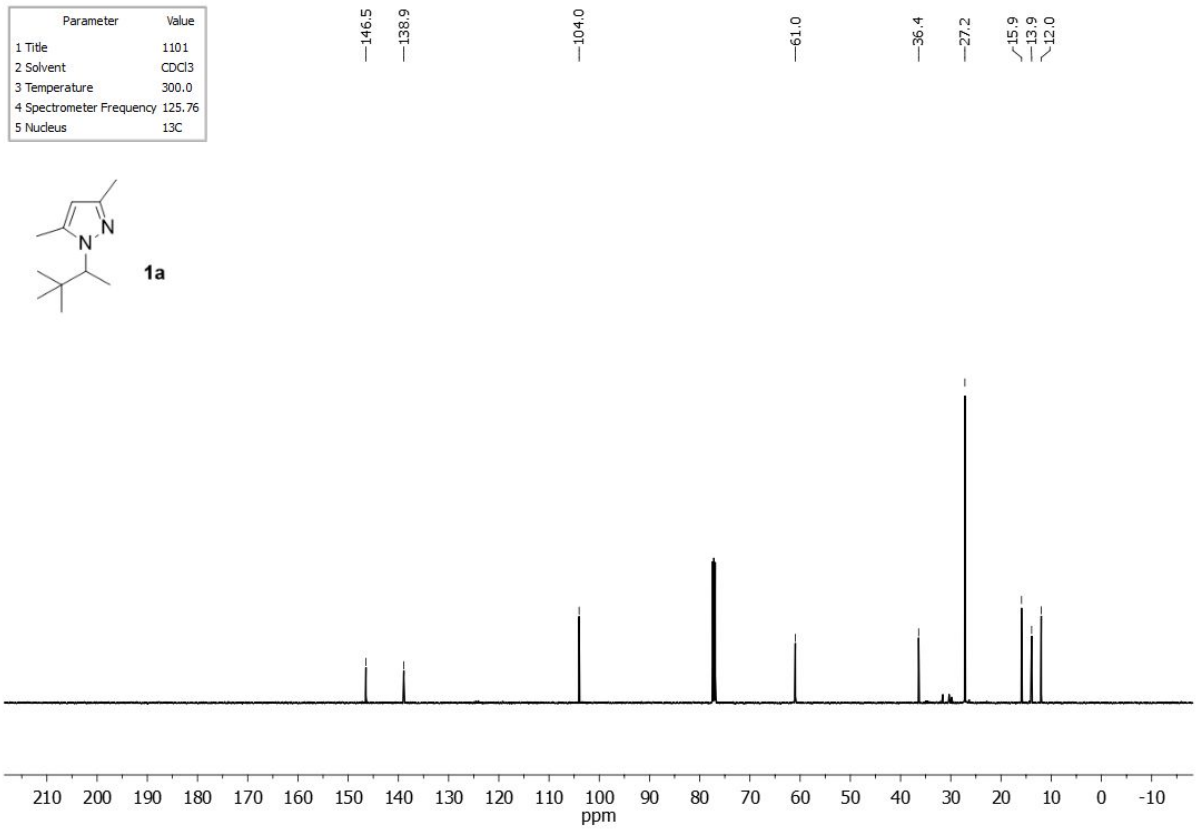

3,5-Dimethyl-1-(2,4,4-trimethylpentan-2-yl)-1*H*-pyrazole, **1b**.

Parameter	Value
1 Solvent	CDCl3
2 Temperature	300.0
3 Spectrometer Frequency	500.13
4 Nucleus	1H

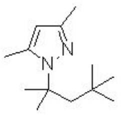

1b

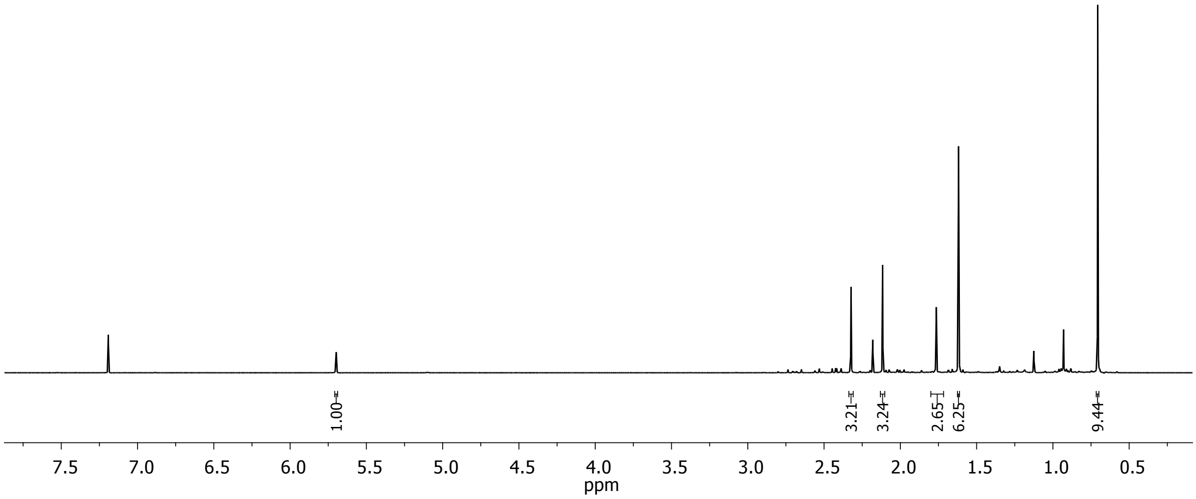

Parameter	Value
1 Solvent	CDCl3
2 Temperature	300.0
3 Spectrometer Frequency	500.13
4 Nucleus	1H

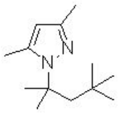

1b

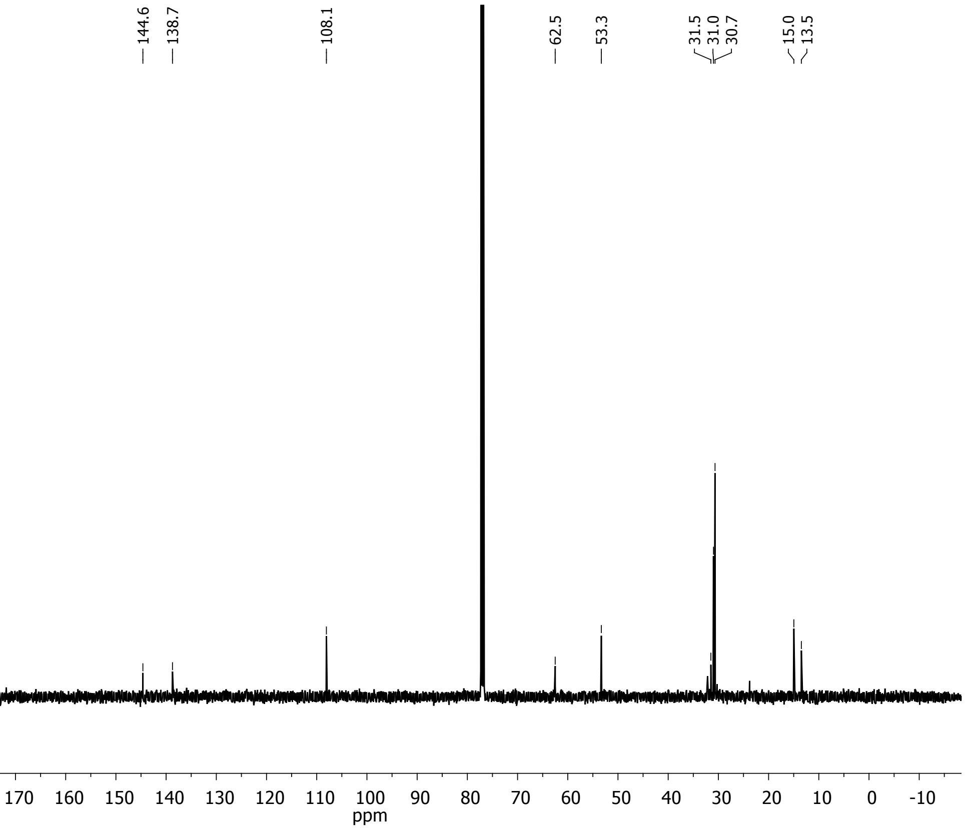

3-(2-(3,5-Dimethyl-1*H*-pyrazol-1-yl)ethyl)-1*H*-indole, **1c**.

Parameter	Value
1 Title	1103f
2 Solvent	CDCl3
3 Temperature	300.0
4 Spectrometer Frequency	500.13
5 Nucleus	1H

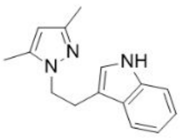

1c

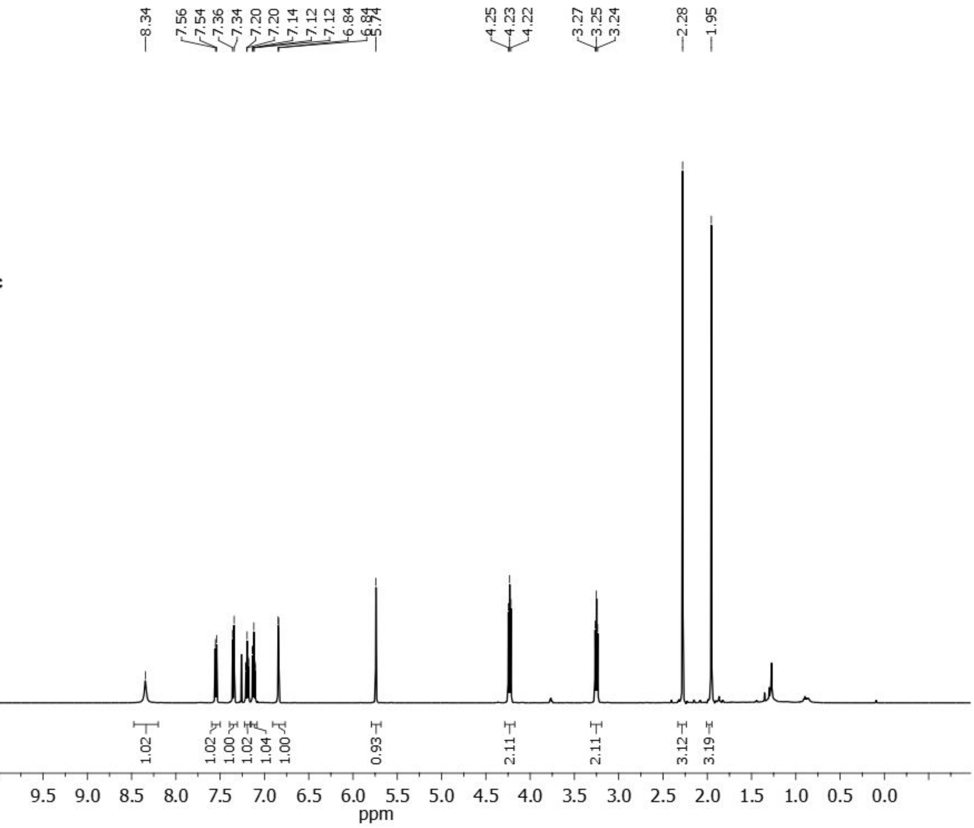

Parameter	Value
1 Title	1103f
2 Solvent	CDCl3
3 Temperature	300.0
4 Spectrometer Frequency	125.76
5 Nucleus	13C

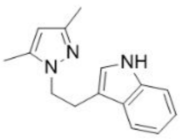

1c

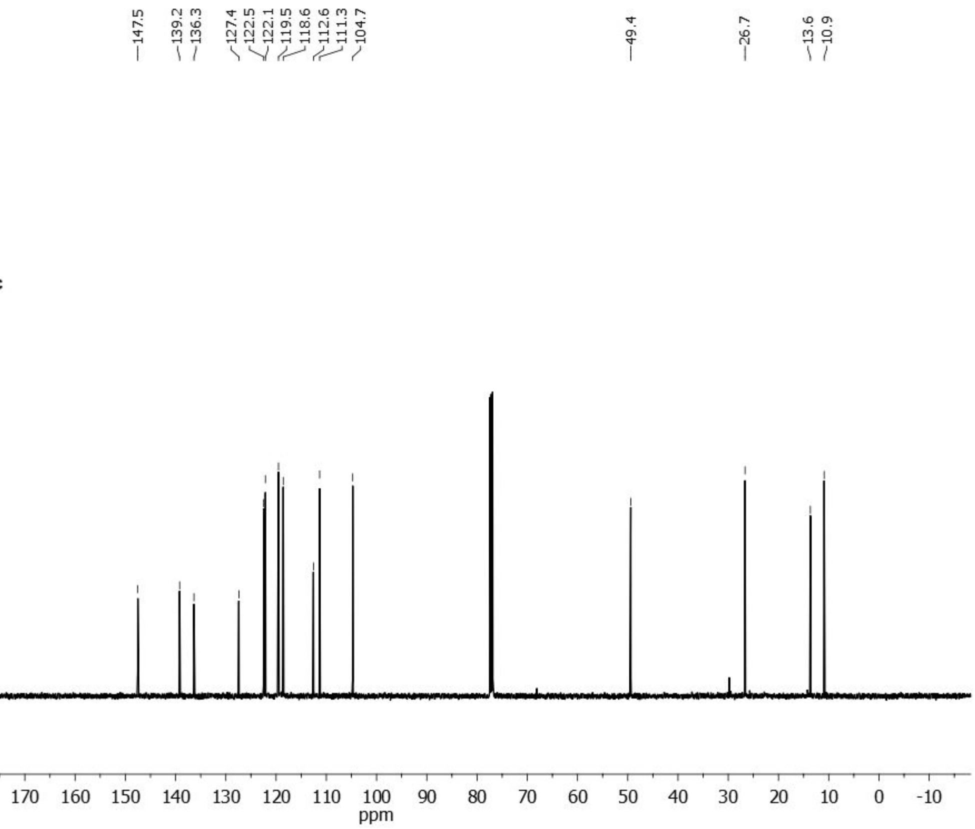

3,5-Dimethyl-1-(*tert*-pentyl)-1*H*-pyrazole, **1d**.

Parameter	Value
1 Solvent	CDCl3
2 Temperature	300.0
3 Spectrometer Frequency	500.13
4 Nucleus	1H

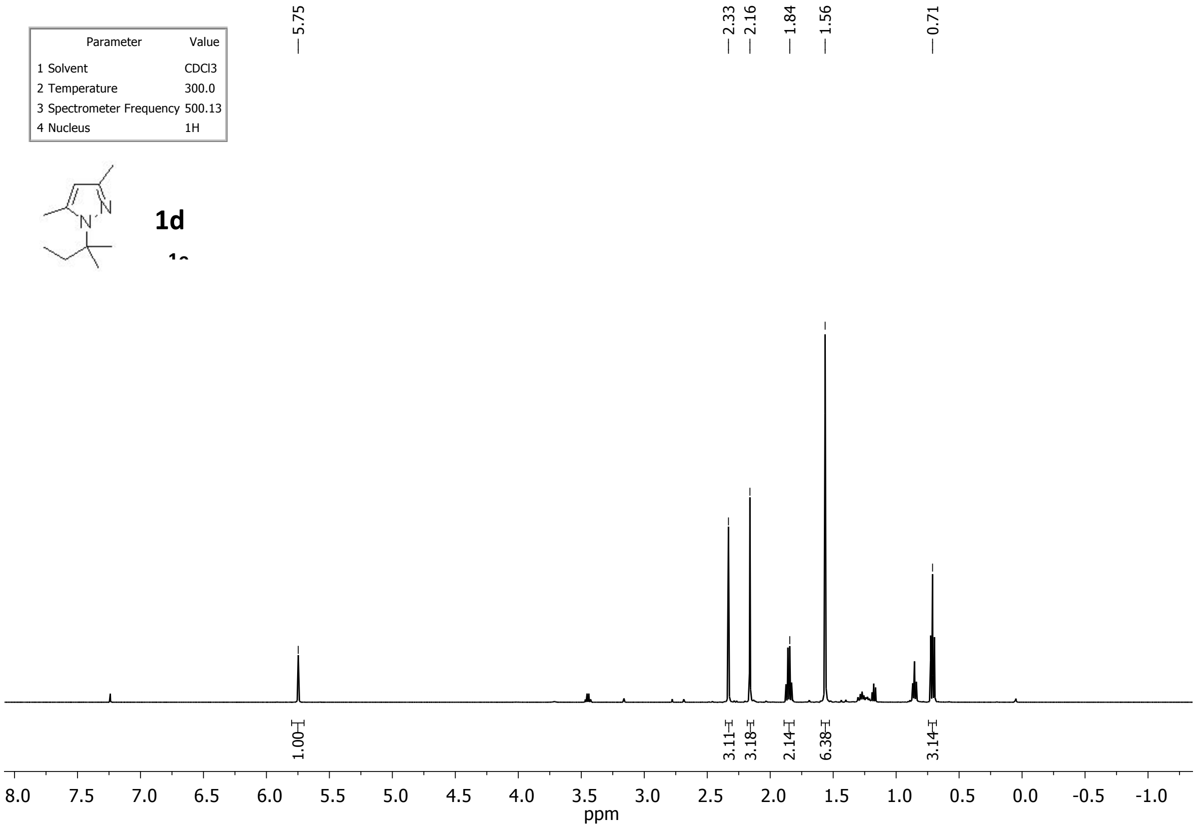

Parameter	Value
1 Solvent	CDCl3
2 Temperature	300.0
3 Spectrometer Frequency	125.77
4 Nucleus	13C

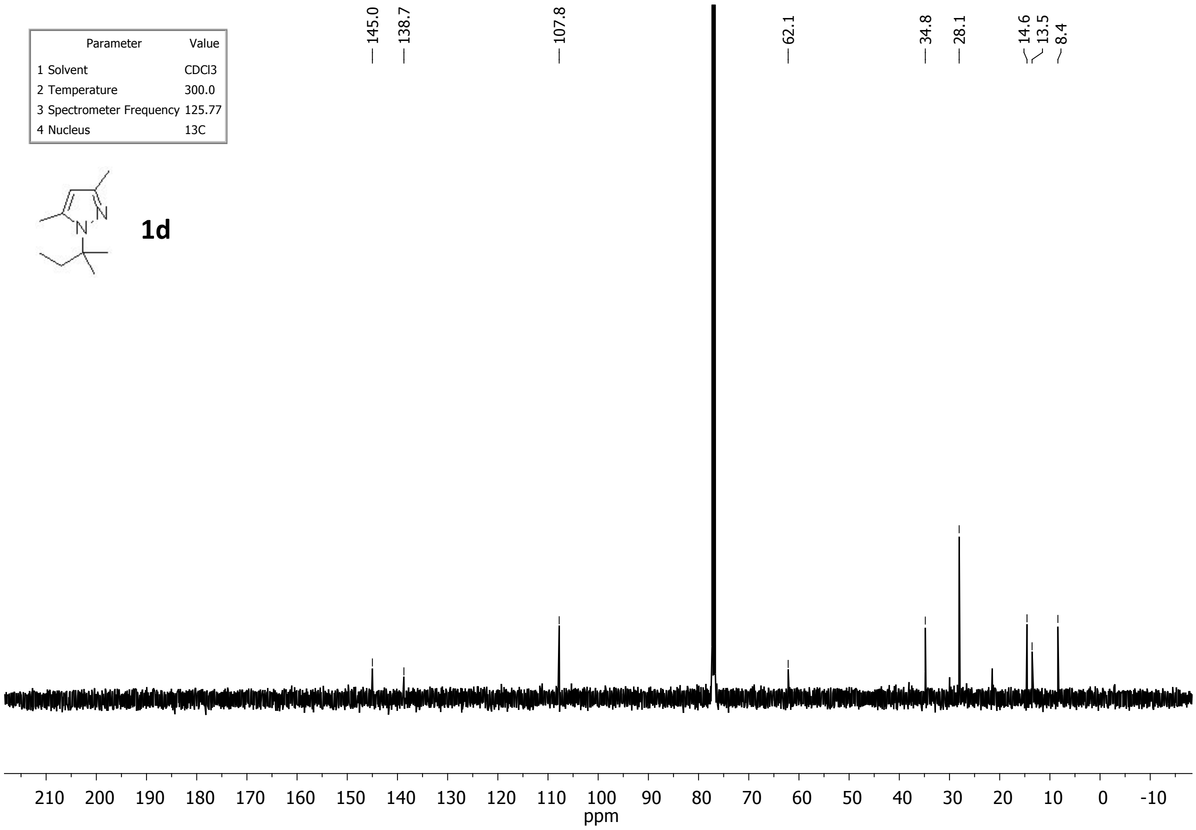

1-Dodecyl-3,5-dimethyl-1*H*-pyrazole, **1e**.

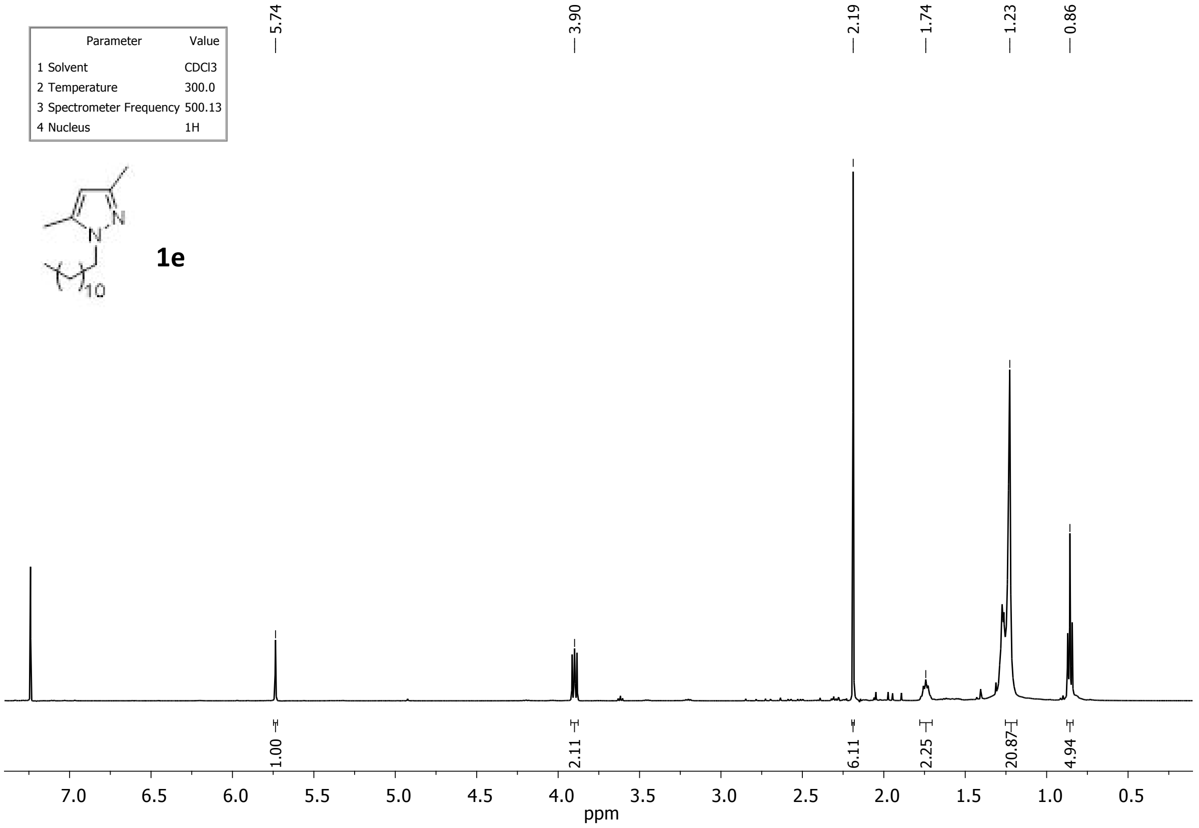

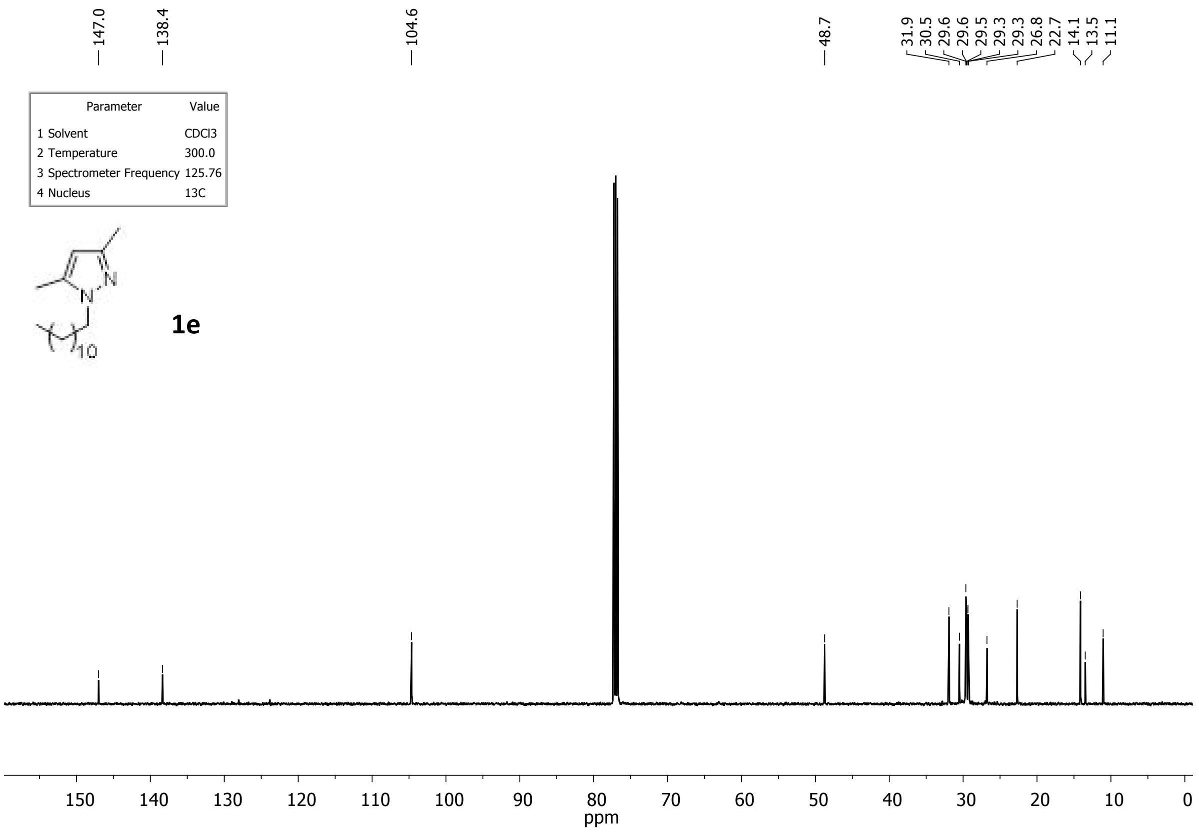

6-(3,5-Dimethyl-1*H*-pyrazol-1-yl)-2-methylheptan-2-ol, **1g**.

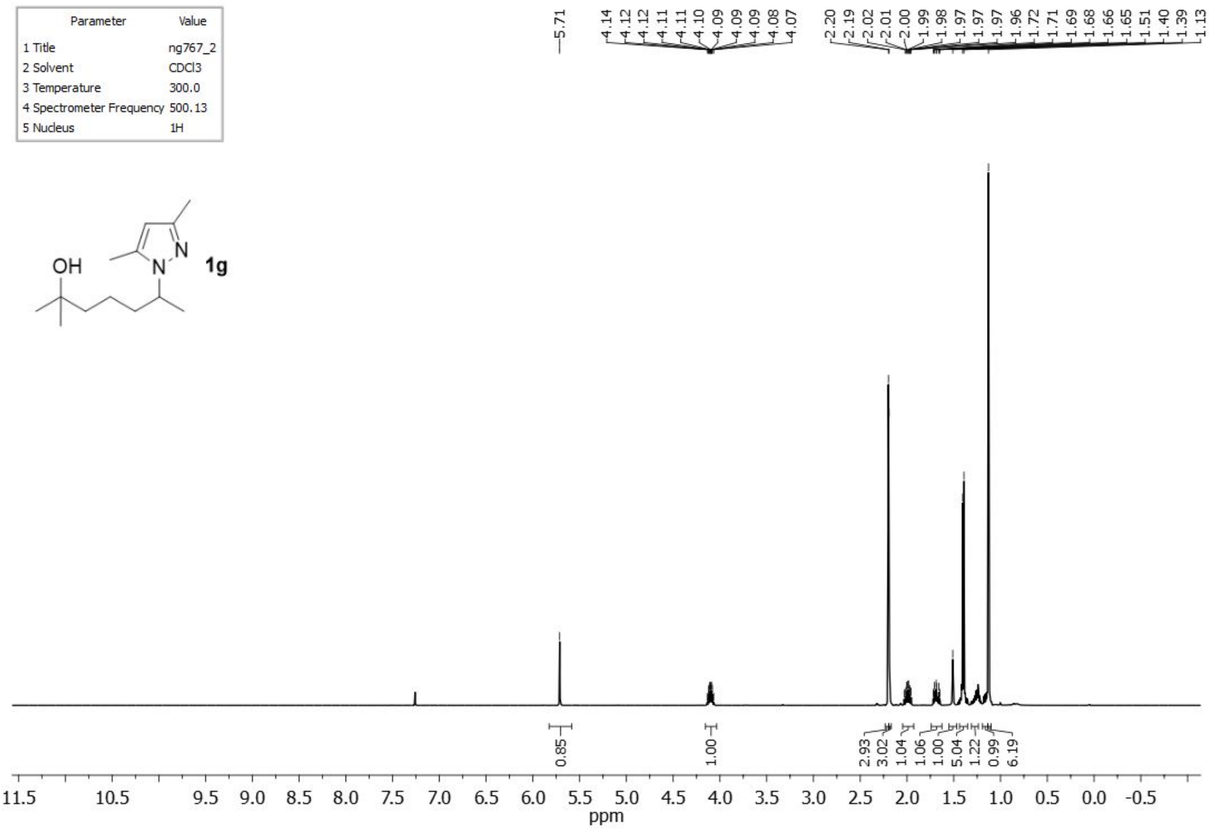

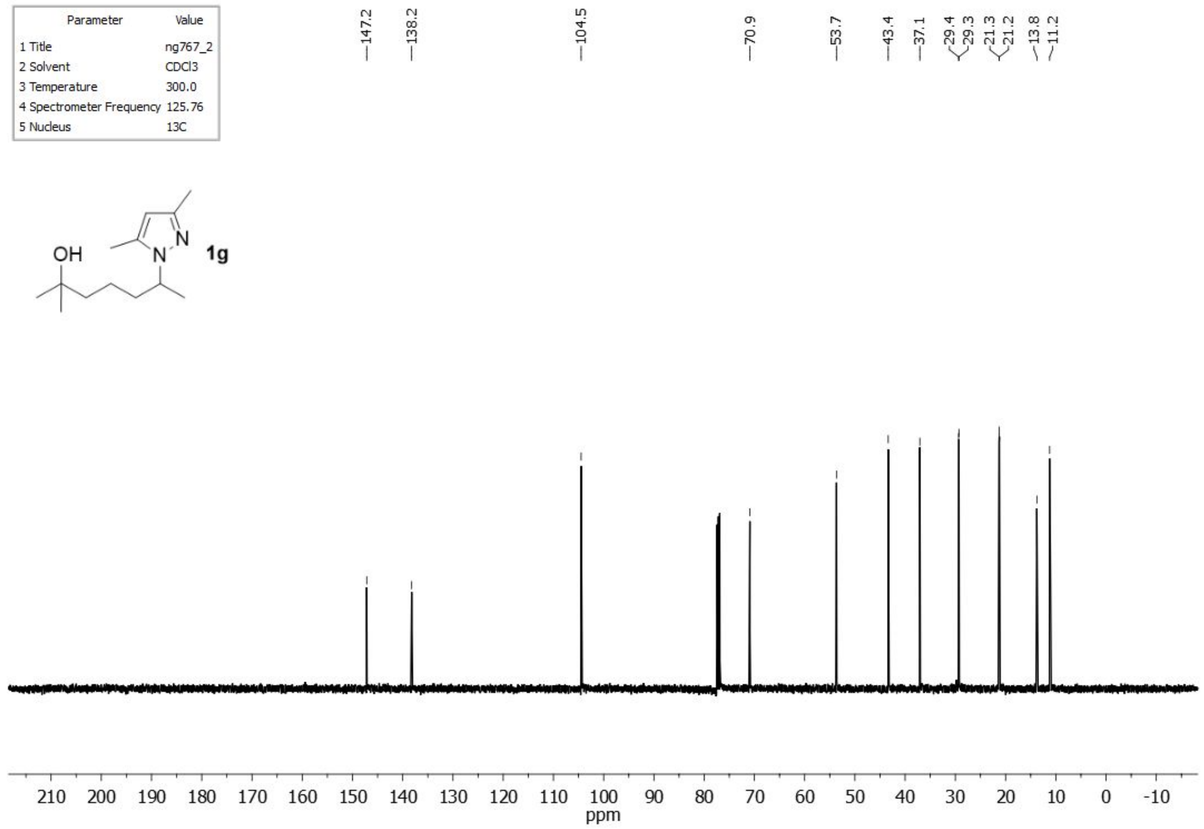

Ethyl 2-(3,5-dimethyl-1*H*-pyrazol-1-yl)-3-phenylpropanoate, **1j**.

Parameter	Value
1 Title	1095
2 Solvent	CDCl3
3 Temperature	300.0
4 Spectrometer Frequency	500.13
5 Nucleus	1H

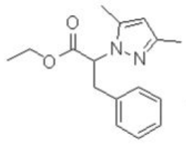

1j

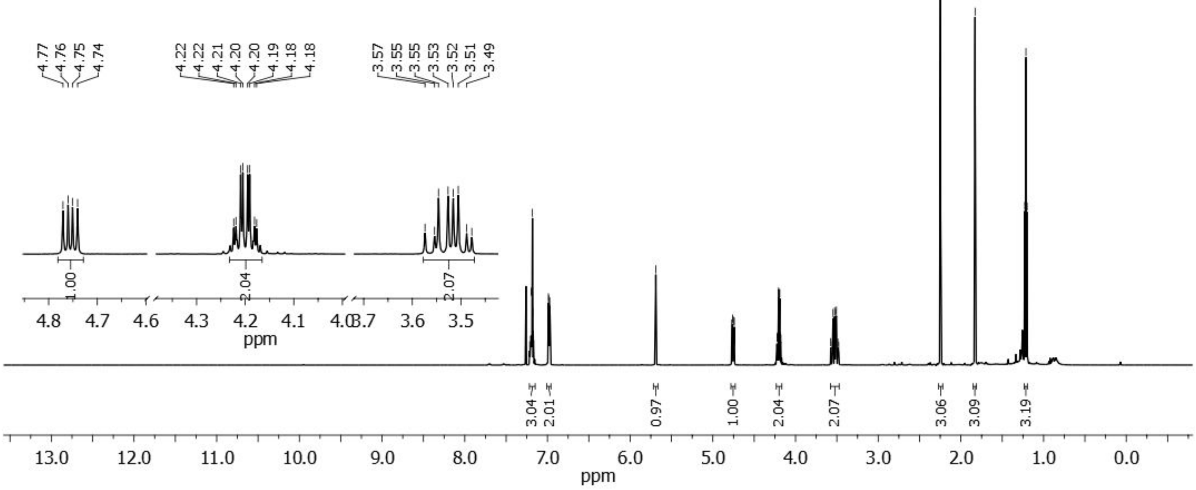

Parameter	Value
1 Title	1095_C
2 Solvent	CDCl3
3 Temperature	301.2
4 Spectrometer Frequency	75.47
5 Nucleus	13C

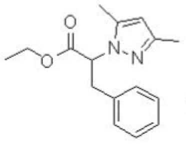

1j

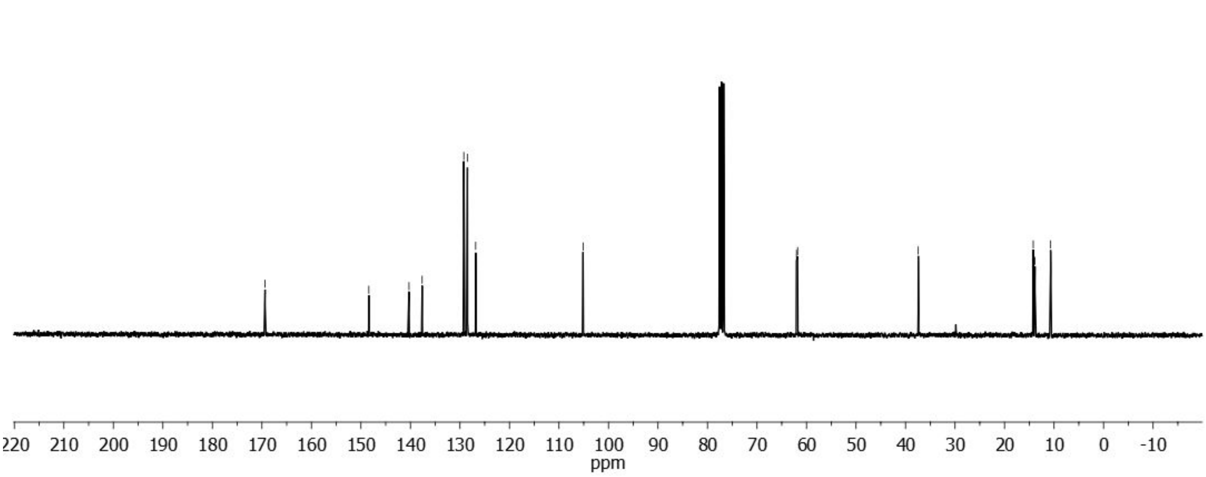

1-(Bicyclo[2.2.1]heptan-2-yl)-3,5-dimethyl-1H-pyrazole, **1k.**

Parameter	Value
1 Solvent	CDCl3
2 Temperature	300.0
3 Spectrometer Frequency	500.13
4 Nucleus	1H

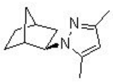

1k

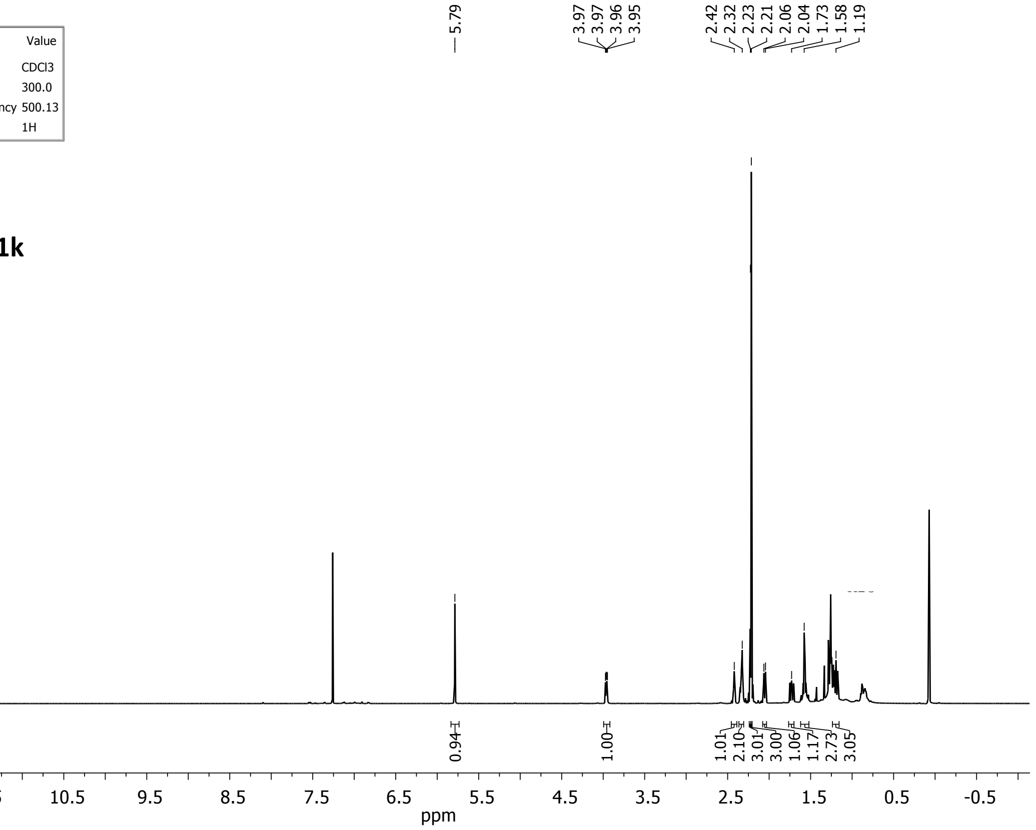

Parameter	Value
1 Solvent	CDCl3
2 Temperature	300.0
3 Spectrometer Frequency	125.76
4 Nucleus	13C

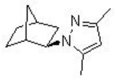

1k

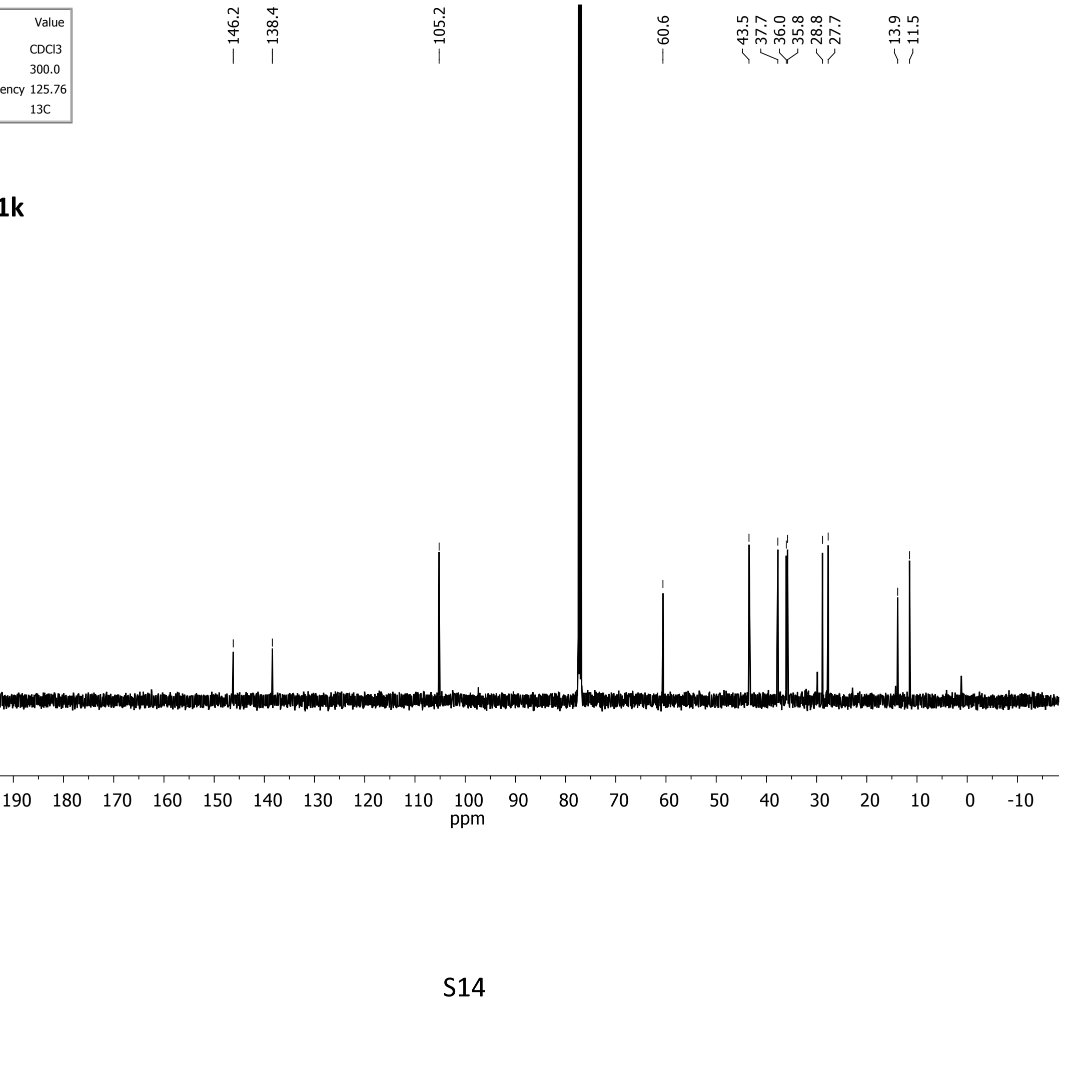

3,5-Dimethyl-1-(naphthalen-2-yl)-1*H*-pyrazole, **1o**.

Parameter	Value
1 Solvent	CDCl3
2 Temperature	300.0
3 Spectrometer Frequency	500.13
4 Nucleus	1H

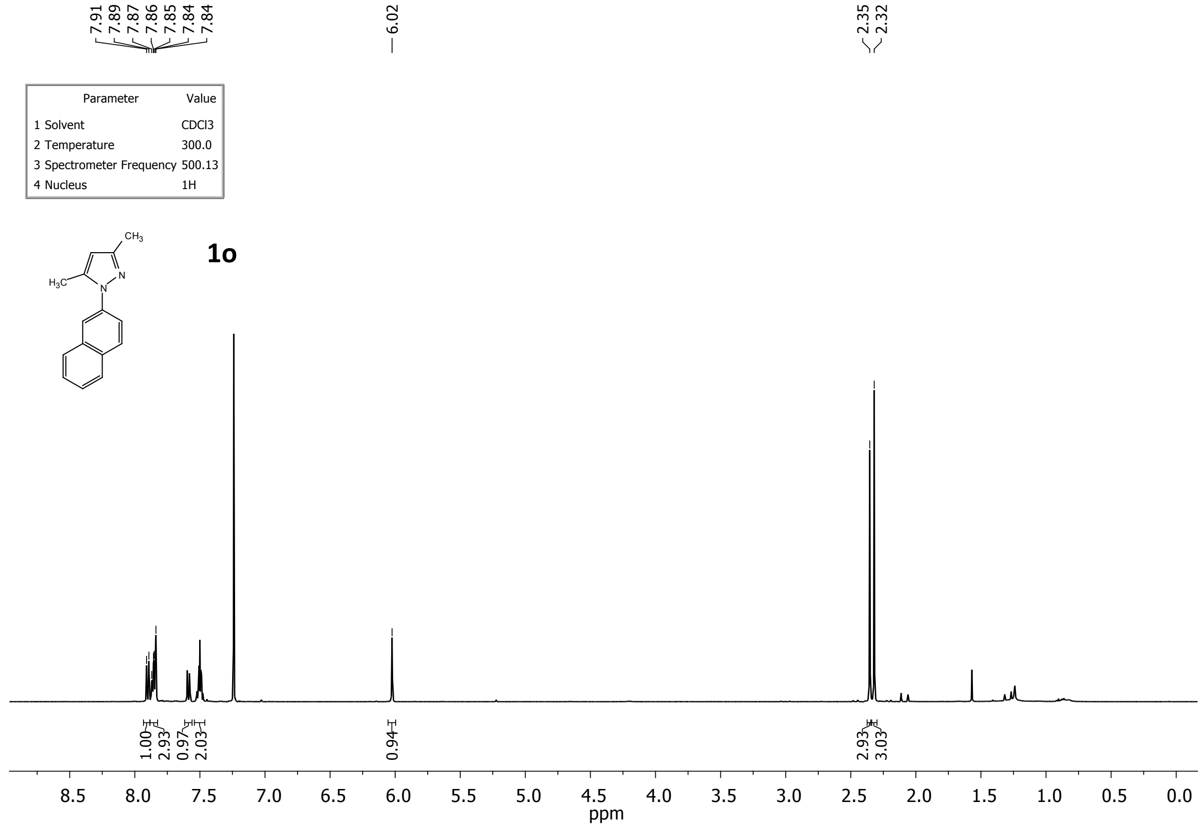

Parameter	Value
1 Solvent	CDCl3
2 Temperature	300.0
3 Spectrometer Frequency	125.76
4 Nucleus	13C

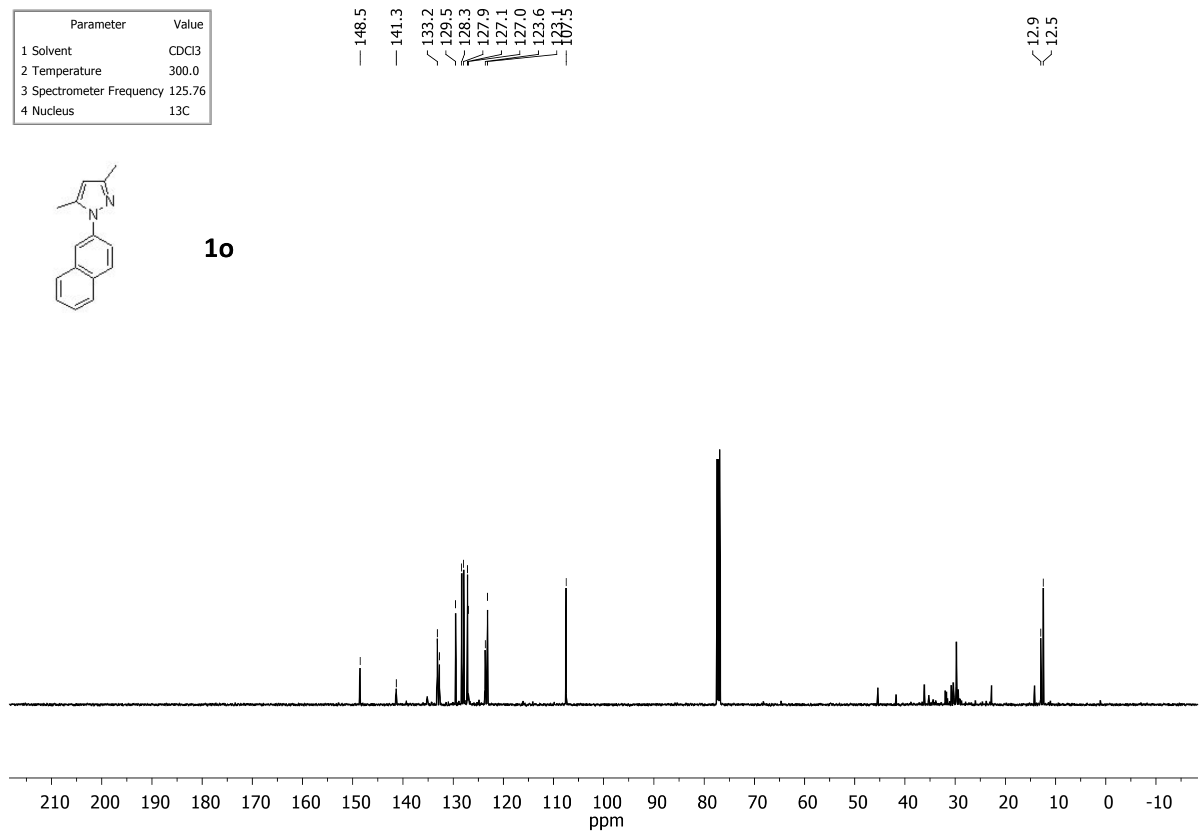

1-(5-Bromo-2-methylphenyl)-3,5-dimethyl-1*H*-pyrazole, **1p**.

Parameter	Value
1 Title	1099
2 Solvent	CDCl3
3 Temperature	300.0
4 Spectrometer Frequency	500.13
5 Nucleus	1H

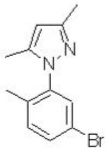

1p

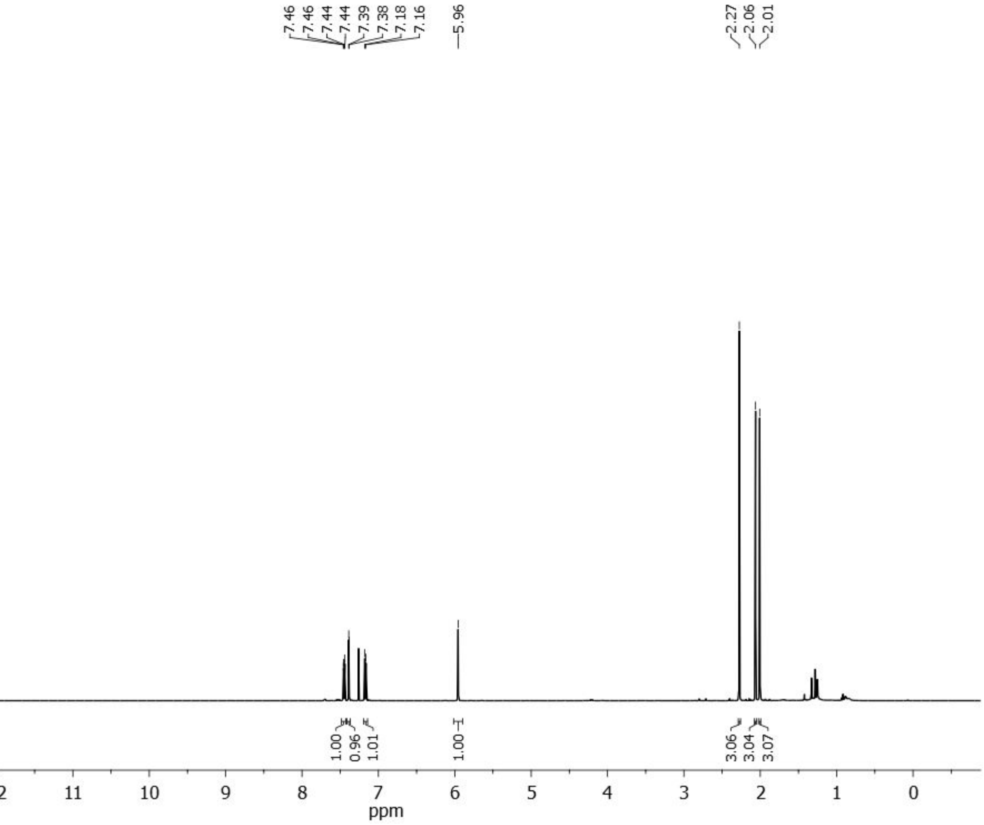

Parameter	Value
1 Title	1099
2 Solvent	CDCl3
3 Temperature	300.0
4 Spectrometer Frequency	125.76
5 Nucleus	13C

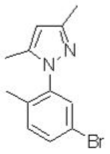

1p

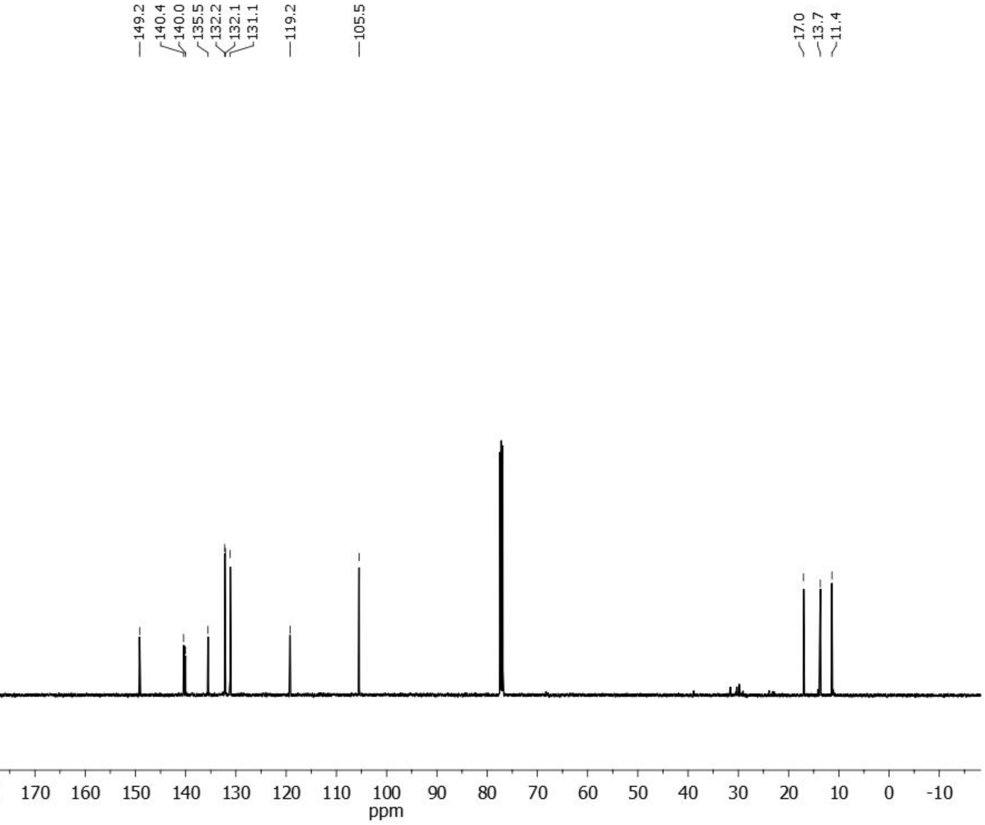

3,4,5-Trimethyl-1-(2,4,4-trimethylpentan-2-yl)-1*H*-pyrazole, **2b**.

Parameter	Value
1 Solvent	CDCl3
2 Temperature	300.0
3 Spectrometer Frequency	500.13
4 Nucleus	1H

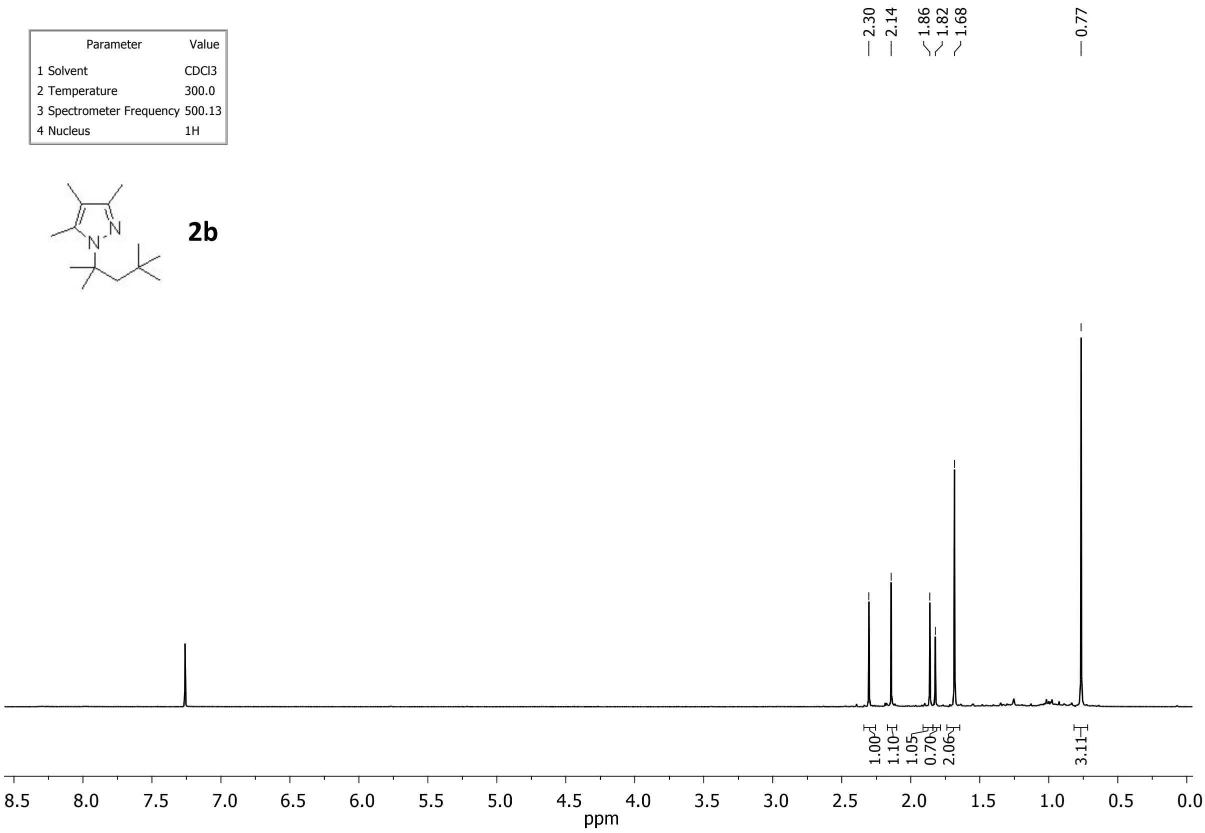

Parameter	Value
1 Solvent	CDCl3
2 Temperature	300.0
3 Spectrometer Frequency	125.76
4 Nucleus	13C

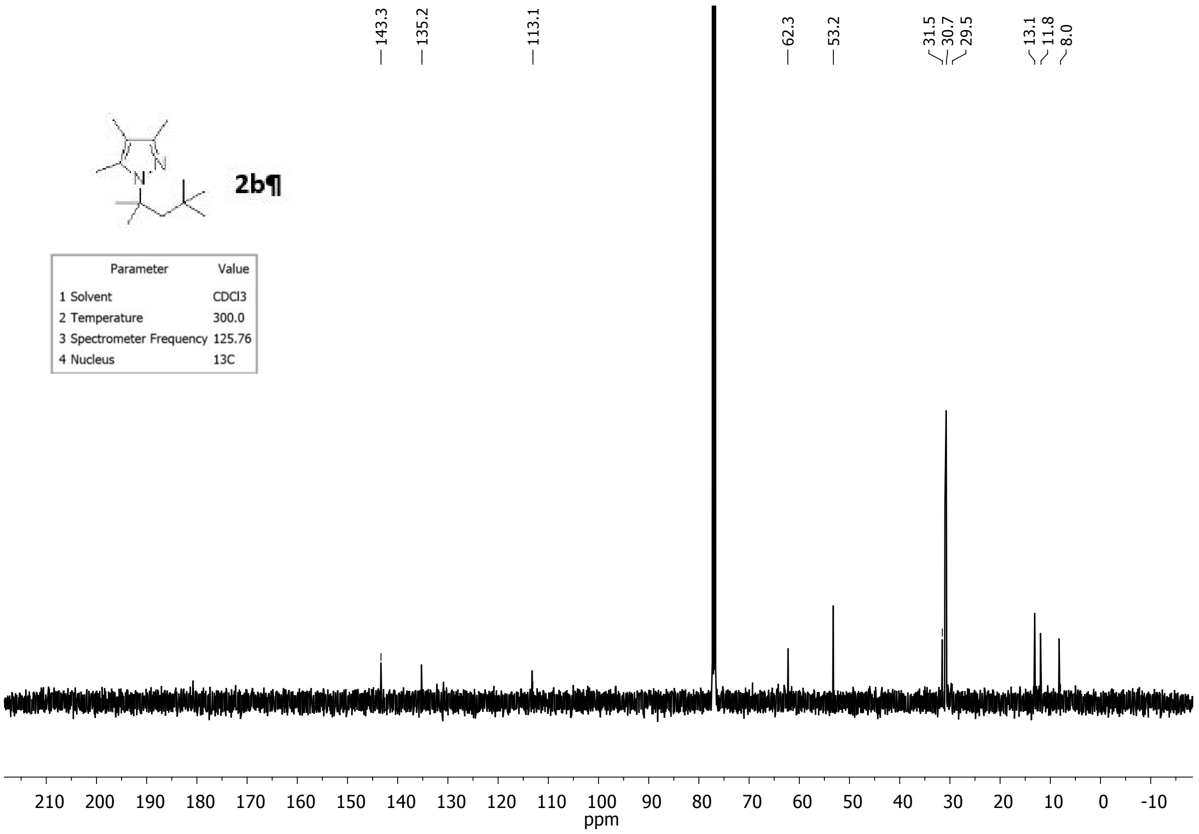

3,5-Diethyl-1-(2,4,4-trimethylpentan-2-yl)-1*H*-pyrazole, **3b**.

Parameter	Value
1 Solvent	CDCl3
2 Temperature	300.0
3 Spectrometer Frequency	500.13
4 Nucleus	1H

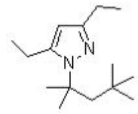

3b

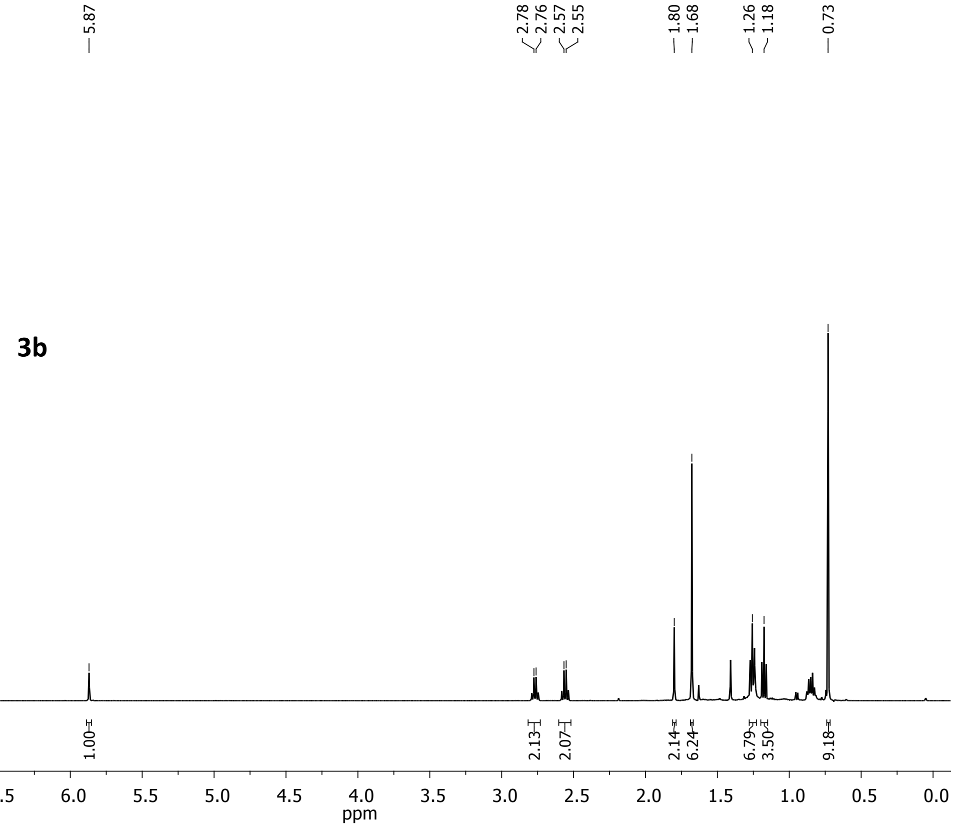

Parameter	Value
1 Solvent	CDCl3
2 Temperature	300.0
3 Spectrometer Frequency	125.76
4 Nucleus	13C

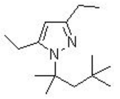

3b

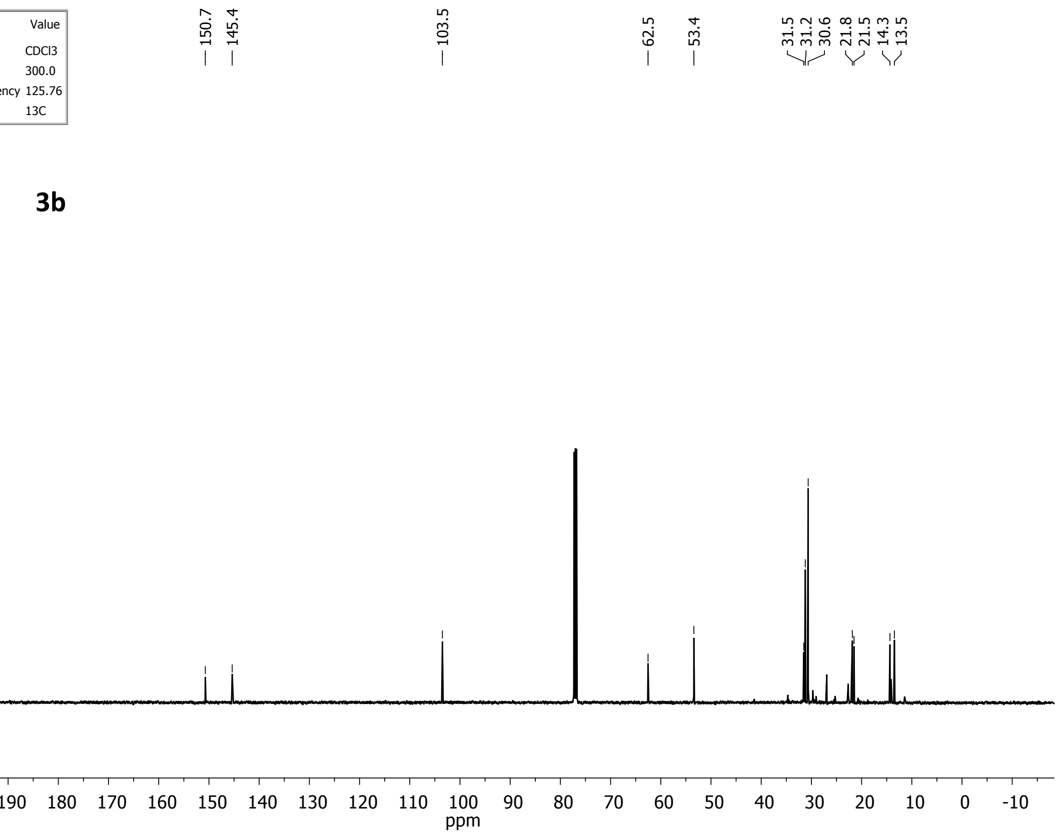

4-Ethyl-3,5-dimethyl-1-(2,4,4-trimethylpentan-2-yl)-1H-pyrazole, **4b**.

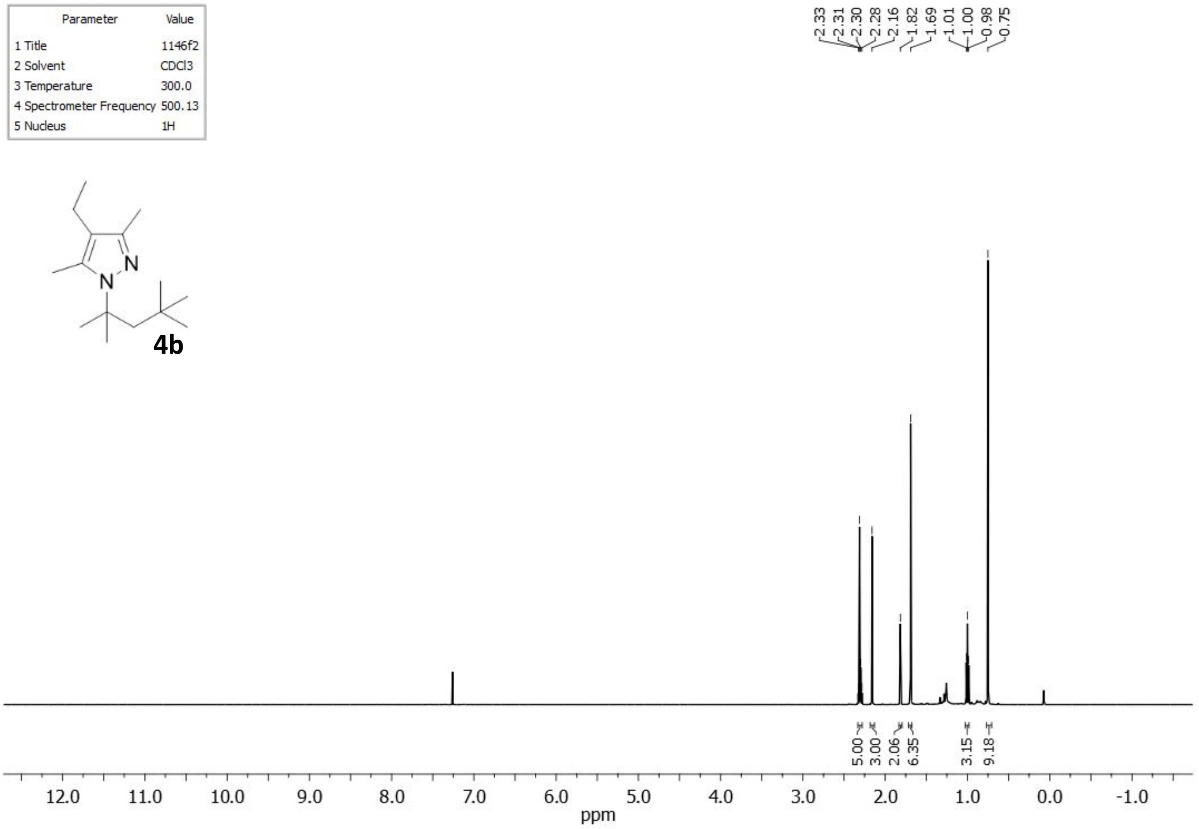

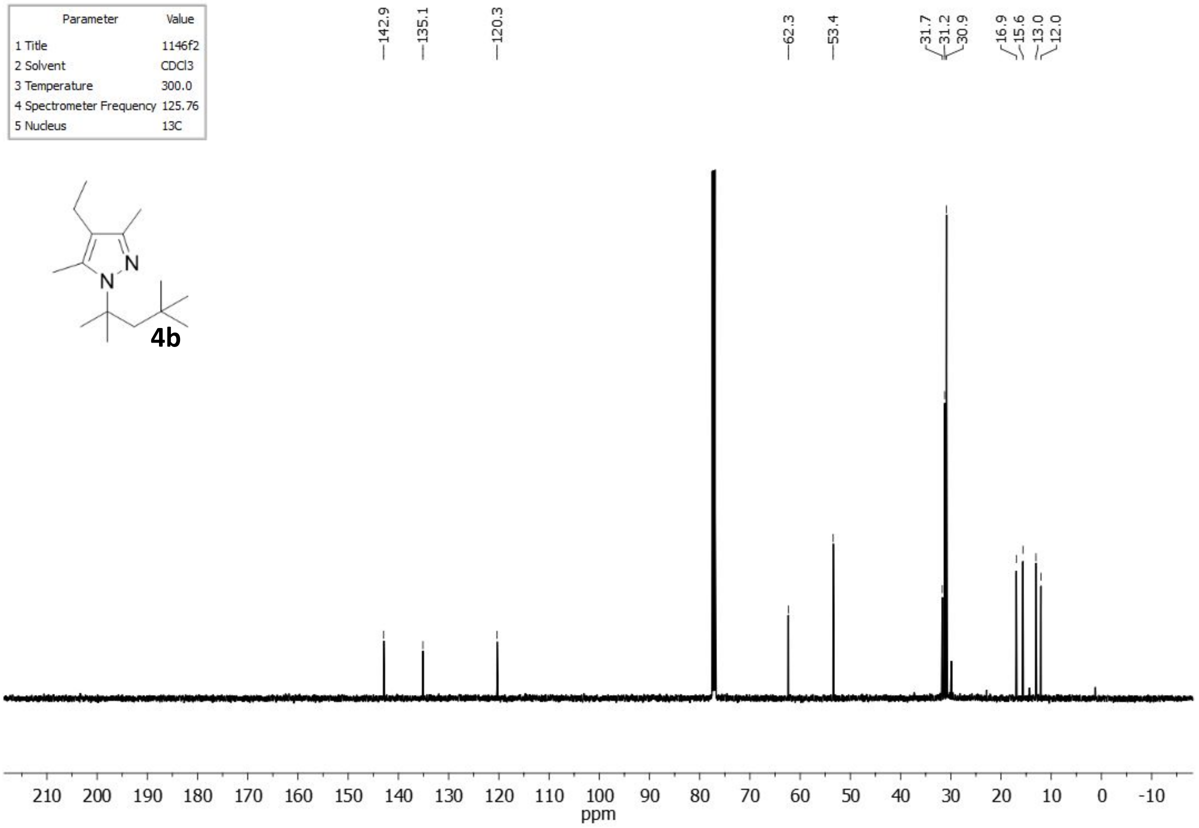

3-Methyl-5-phenyl-1-(2,4,4-trimethylpentan-2-yl)-1H-pyrazole, **5b**.

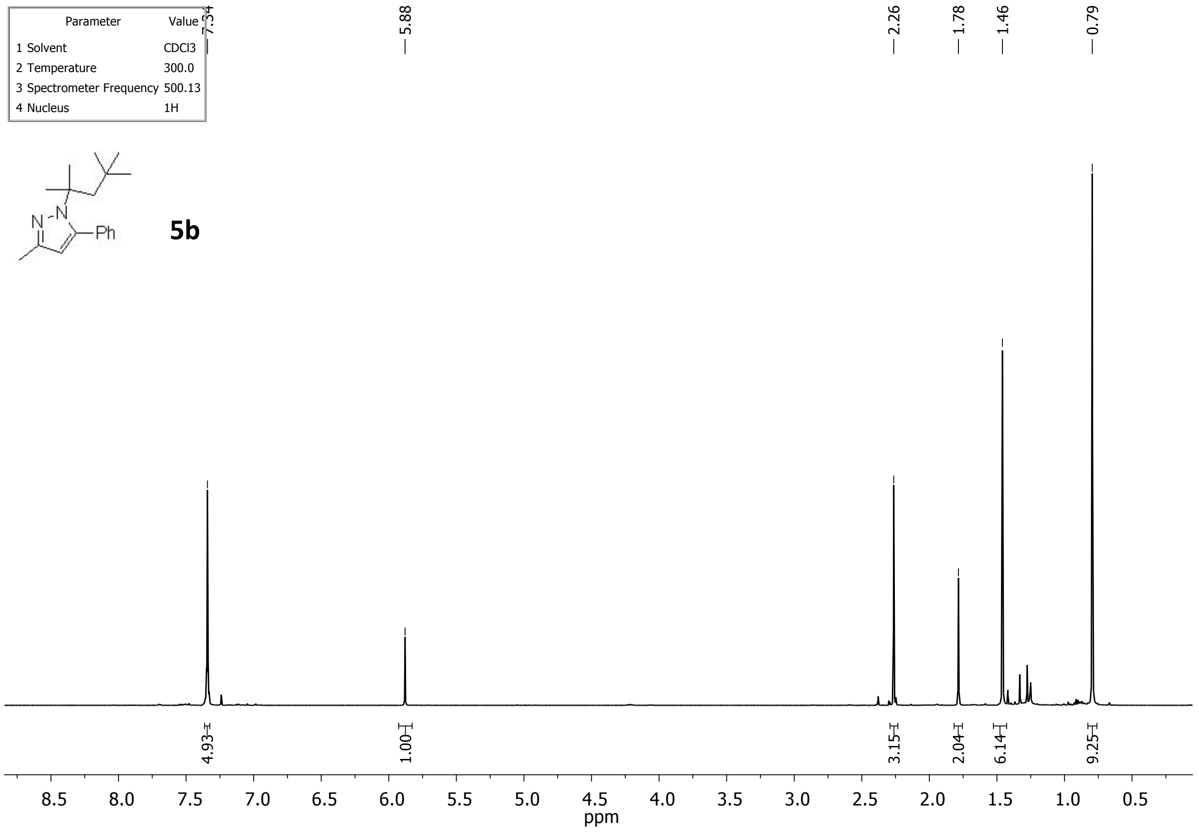

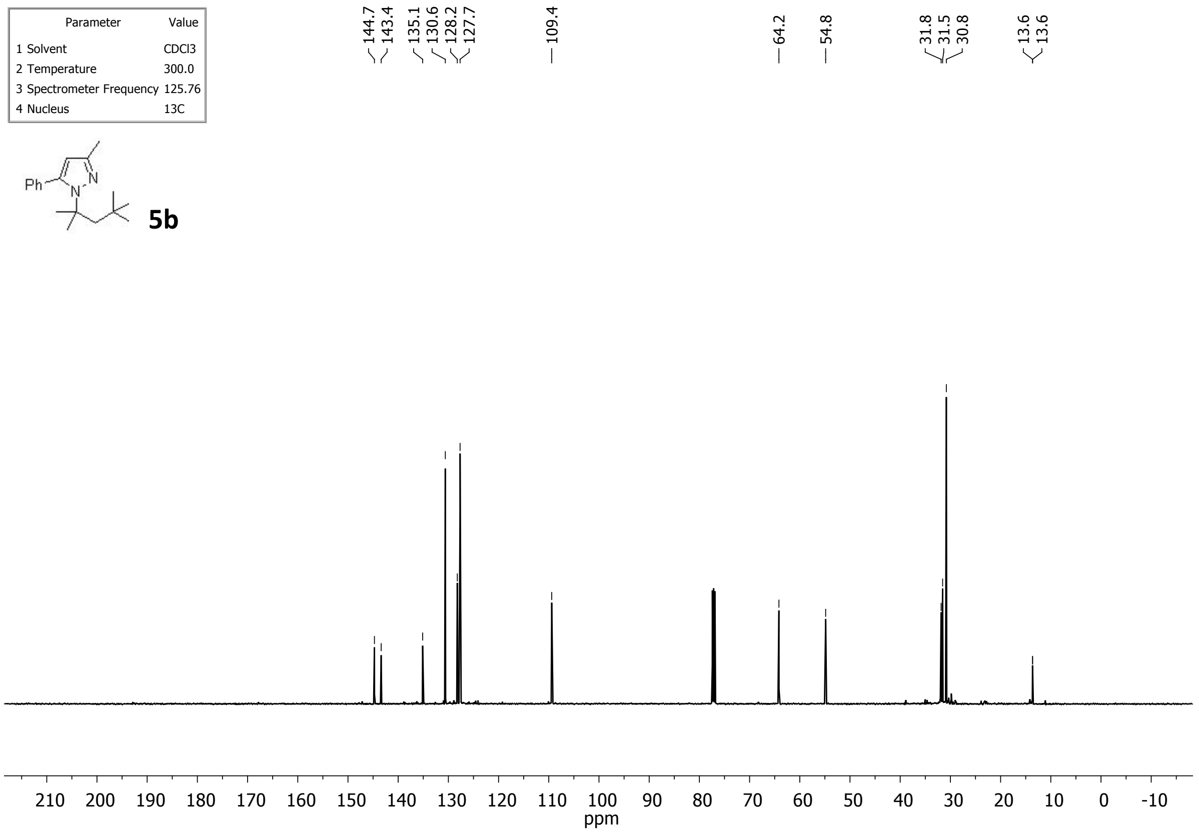

5-Isobutyl-3-methyl-1-(2,4,4-trimethylpentan-2-yl)-1*H*-pyrazole, **6b.**

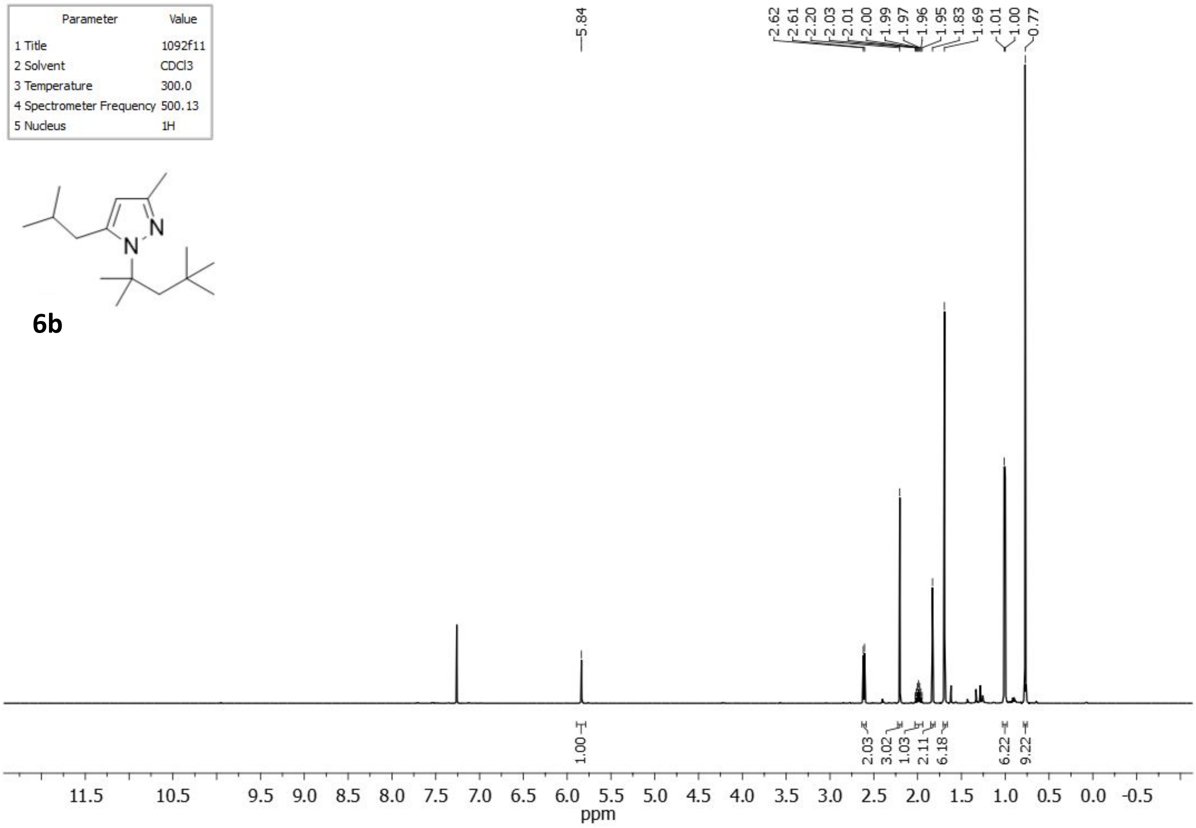

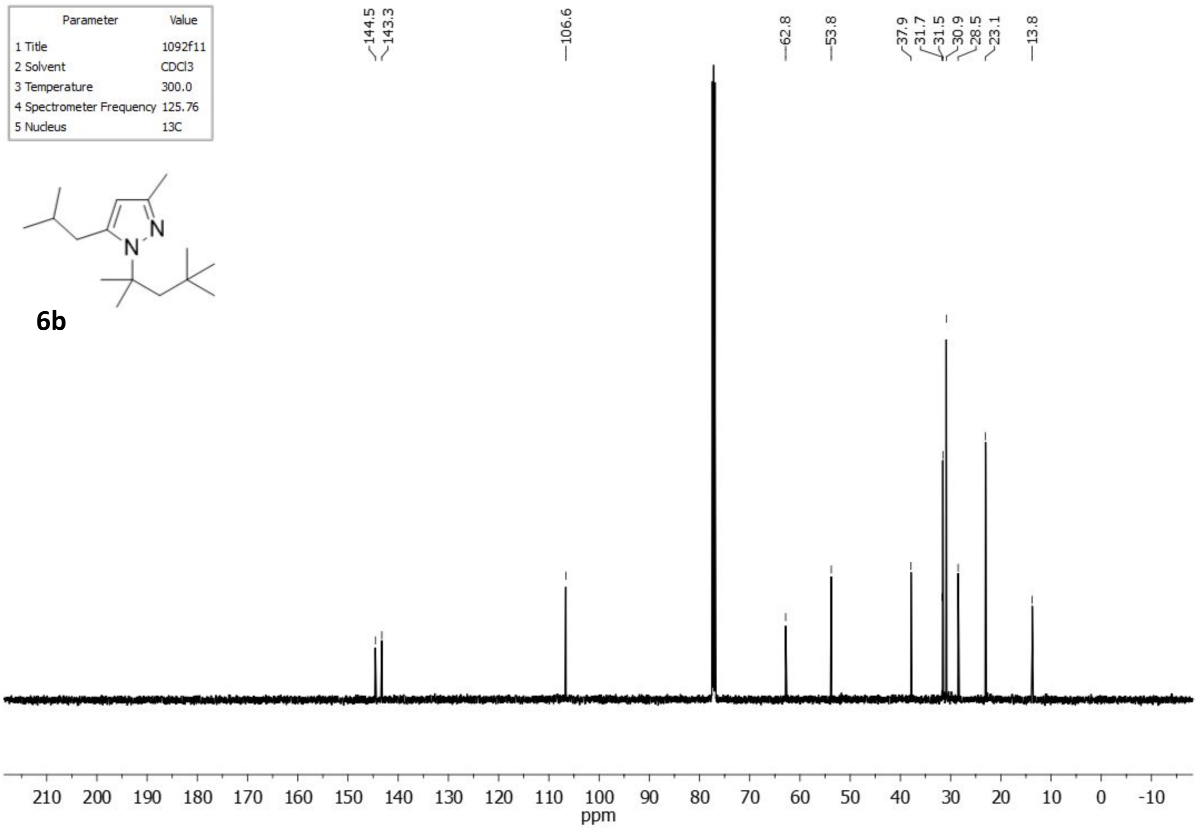

Supplement: Supplementary file 1 — jo1c00606_si_001.pdf [file jo1c00606_si_001.pdf]
